# Inflammatory Cytokine-Induced HIF-1 Activation Promotes Epithelial–Mesenchymal Transition in Endometrial Epithelial Cells

**DOI:** 10.3390/biomedicines11010210

**Published:** 2023-01-14

**Authors:** Yoshiko Hashimoto, Tomoko Tsuzuki-Nakao, Naoko Kida, Yoshiyuki Matsuo, Tetsuo Maruyama, Hidetaka Okada, Kiichi Hirota

**Affiliations:** 1 Department of Obstetrics and Gynecology, Kansai Medical University, 2-5-1 Shinmachi, Hirakata 573-1191, Japan; 2 Department of Human Stress Response Science, Institute of Biomedical Science, Kansai Medical University, 2-5-1 Shinmachi, Hirakata 573-1010, Japan; 3 Department of Obstetrics and Gynecology, Keio University School of Medicine, Tokyo 160-8582, Japan

**Keywords:** endometrium, epithelial cells, Hypoxia-Inducible Factor 1, inflammation, epithelial–mesenchymal transition

## Abstract

The endometrium undergoes repeated proliferation and shedding during the menstrual cycle. Significant changes to this environment include fluctuations in the partial pressure of oxygen, exposure to a high-cytokine environment associated with intrauterine infection, and inflammation. Chronic endometritis is a condition wherein mild inflammation persists in the endometrium and is one of the causes of implantation failure and miscarriage in early pregnancy. It is thought that the invasion of embryos into the endometrium requires epithelial–mesenchymal transition (EMT)-associated changes in the endometrial epithelium. However, the effects of inflammation on the endometrium remain poorly understood. In this study, we investigated the effects of the intrauterine oxygen environment, hypoxia-inducible factor (HIF), and inflammation on the differentiation and function of endometrial epithelial cells. We elucidated the ways in which inflammatory cytokines affect HIF activity and EMT in an immortalized cell line (EM-E6/E7/TERT) derived from endometrial epithelium. Pro-inflammatory cytokines caused significant accumulation of HIF-1α protein, increased HIF-1α mRNA levels, and enhanced hypoxia-induced accumulation of HIF-1α protein. The combined effect of inflammatory cytokines and hypoxia increased the expression of EMT-inducing factors and upregulated cell migration. Our findings indicate that pro-inflammatory factors, including cytokines and LPS, work synergistically with hypoxia to activate HIF-1 and promote EMT in endometrial epithelial cells.

## 1. Introduction

The endometrium undergoes repeated proliferation and shedding during the menstrual cycle. Important changes to this environment include fluctuations in the partial pressure of oxygen, exposure to a high-cytokine environment associated with intrauterine infection, and inflammation. Numerous studies support the idea that hypoxia-inducible factors (HIFs) play a key role in the dynamics of endometrial shedding during menstruation owing to considerable changes in the oxygen partial pressure in tissue [1,2,3,4,5]. Pathways regulating HIF activation, independent of changes in oxygen partial pressure, have also been identified [6,7,8]. Inflammation plays essential roles as prostaglandins and inflammatory mediators, such as tumor necrosis factor-α (TNFα) and lipopolysaccharide (LPS), induce HIF-1α activation under non-hypoxic conditions [9,10,11]. The regulatory pathway of HIF activity by these inflammatory agents, together with the regulation of activation by changes in the partial pressure of oxygen, explain the diverse roles of HIF. HIF-1 activation by pro-inflammatory cytokines has been documented in various cells, including macrophages, monocytes, hepatocytes, and renal cells [12,13,14]. The regulation of HIF activity through these inflammatory agents along with changes in oxygen partial pressure—including hypoxia—hint at the diverse roles of HIF. However, few studies have reported HIF activity in the endometrium under inflammatory conditions.

Chronic endometritis is a condition in which mild inflammation persists in the endometrium [15]. It is considered one of the causes of implantation failure and miscarriage in early pregnancy [16,17]. Chronic endometritis is a reactive process caused by bacterial infection, among other factors, and occurs in 2.8–56.8% of infertile women [18,19,20,21]. This broad range of the frequency of occurrence is likely due to a lack of uniform diagnostic criteria, which stems from a lack of knowledge regarding its pathogenesis. In addition to chronic endometritis, endometriosis is also one of the benign and chronic inflammatory diseases associated with the endometrium wherein endometrium-like tissues are present outside the uterus. Endometriosis has unique characteristics in that endometriotic cells share some features with cancer cells, such as invasiveness and metastatic potential. Several studies have indicated that the epithelial–mesenchymal transition (EMT) is involved in the pathogenesis of endometriosis [22,23,24]. In the EMT, epithelial traits are lost, mesenchymal features develop, and the potential for migration and invasion is boosted. E-cadherin deficiency is closely associated with cancer invasion and poor prognosis in patients with endometrial cancer [25]. A fertility-preserving treatment can be taken into consideration in some patients with early-stage endometrial cancer, since it occurs in up to 29% of women before 40 years of age [26,27]. The prediction of prognosis is an important component of the choice of treatment, and one of the established prognostic factors is the mutation of genes including *PTEN*, *POLE*, and *TP53* [28]. On the other hand, several investigations found genetic anomalies in tumor-suppressing genes such as *TP53* and *PTEN* in endometriosis, a chronic inflammatory disease [29,30]. Inflammatory cells and cytokines promote the production of reactive oxygen species, which may, in turn, induce DNA damage and gene mutations. Deletions or mutations in tumor-suppressor genes such as *TP53* and *PTEN* have been reported to result in the activation of HIFs even under non-hypoxic conditions [7,31]. High expression of HIF-1α has also been shown to predict decreased survival in endometrial cancer [32]. The induction of the EMT and increased expression of HIFs have recently been reported in endometriotic lesions, which consist mainly of stromal and glandular epithelial cells [33,34]. To elucidate potential therapeutic targets for diseases of the endometrium, it is imperative that we evaluate the involvement of endometrial cells in the EMT and that of inflammatory agents under hypoxia.

This study investigates the interaction between the intrauterine oxygen environment, HIF activation, and inflammation, and evaluates the roles of these interactions in the differentiation and function of endometrial epithelial cells. We used an immortalized cell line derived from endometrial epithelium to examine how inflammatory cytokines affect the activity of the transcription factor HIF and its consequent impact on the EMT.

## 2. Materials and Methods

### 2.1. Reagents

Recombinant human TNFα and IL-1β were purchased from PeproTech (Cranbury, NJ, USA), and LPS (from E. coli O111:B4) was purchased from InvivoGen (Toulouse, France). N-(2-methoxy-2-oxoacetyl) glycine methyl ester (DMOG; Sigma-Aldrich, St. Louis, MO, USA) and N-acetyl-L-cysteine (NAC; Abcam, Cambridge, UK) were dissolved in ultrapure water, LY294002 (FUJIFILM Wako Pure Chemical Corporation, Osaka, Japan), PD98059 (FUJIFILM), and SC-514 (Sigma-Aldrich) in dimethyl sulfoxide. Echinomycin was purchased from Enzo Life Sciences (Farmingdale, NY, USA). Table A1 provides a list of the reagents and supplies utilized in this study.

### 2.2. Cell Culture

The immortalized human endometrial epithelial cell line EM-E6/E7/TERT was kindly provided by Dr. T. Maruyama (Keio University School of Medicine, Shinjuku, Tokyo, Japan) [35]. Cells were cultured on plastic dishes and maintained in Dulbecco’s modified Eagle’s medium supplemented with 10% fetal calf serum (FCS; HyClone, Logan, UT, USA), 100 U/mL penicillin, and 0.1 mg/mL streptomycin (Nacalai Tesque, Kyoto, Japan) at 37 °C under 5% CO_2_ in a humidified incubator.

### 2.3. Western Blot Analysis

As previously mentioned [36,37], whole-cell lysates were created using lysis solution including RIPA buffer (FUJI-FILM) and a protease inhibitor cocktail (Calbiochem, La Jolla, CA, USA). To create cell pellets, the samples were centrifuged at 10,000× *g*. A Bio-Rad DC Protein Assay was used to measure protein concentrations (Bio-Rad La-boratories, Hercules, CA, USA). Using a Trans-Blot Turbo transfer device, equal amounts of lysates were electrophoresed on a 7.5 or 12% sodium dodecyl sulfate polyacrylamide gel and electro-transferred onto membranes (Bio-Rad). Blocking One solution (Nacalai Tesque) was used to block non-specific binding sites for 20 min. Purified primary antibodies were used to probe the membranes, including mouse monoclonal anti-HIF-1α (1:1000; BD Transduction Laboratories, Tokyo, Japan), rabbit polyclonal anti-HIF-2α (1:1000; Novus Biologicals, Centennial, CO, USA), rabbit monoclonal anti-HIF-1 β(1:1000; Cell Signaling Technology, Danvers, MA, USA), rabbit monoclonal anti-p44/42 MAPK (1:2000), rabbit monoclonal anti-p38 MAPK (1:1000), rabbit monoclonal anti-Phospho-p44/42 MAPK (1:1000), rabbit monoclonal anti-Phospho-p38 MAPK (1:1000; Cell Signaling Technology), and mouse monoclonal anti-β-actin (1:5000; Sigma-Aldrich). Anti-rabbit IgG peroxidase-labeled (1:5000; Cell Signaling Technology) or anti-mouse IgG peroxidase-labeled (1:5000; Cytiva, Marlborough, MA, USA) antibodies were used as secondary antibodies. Enhanced chemi-luminescence and Western-blotting detection agents were used to observe immune complexes (Cytiva).

### 2.4. Semi-Quantitative Reverse Transcriptase-Polymerase Chain Reaction (qRT-PCR)

Total RNA was extracted from the cells using a Maxwell RSC simplyRNA Cells Kit (Promega, Madison, WI, USA). Reverse transcription was carried out in accordance with the manufacturer’s instructions using a first-strand cDNA synthesis kit called ReverTra Ace qPCR RT master mix from TOYOBO in Osaka, Japan. Using a Thunderbird SYBER qPCR mix kit (TOYOBO) and Rotor-Gene QHRM (Qiagen, Hilden, Germany) in accordance with the manufacturer’s guidelines, real-time PCR was carried out. The Ct technique was used to calculate the relative gene expression. The elongation factor (EF)-1, which was utilized as an internal control, was used to standardize Ct readings. The list of primer sets used for RT-PCR is displayed in Table 1.

### 2.5. RNA-Seq

Total RNA was extracted from the cells using a Maxwell RSC simplyRNA Cells Kit (Promega). RNA sequence libraries were prepared using a TruSeq Stranded mRNA Sample Prep Kit and a TruSeq Stranded mRNA Library Preparation Kit (Illu-mina, San Diego, CA, USA) and sequenced in 100 bp paired-end reads on a NovaSeq 6000 platform (Illumina). The raw sequencing data were deposited in the Sequence Read Archive (https://www.ddbj.nig.ac.jp/dra/index-e.html, accessed on 14 July 2022; accession numbers: DRR395305–DRR395313). RNA-Seq was carried out three times. The website protocols.io (dx.doi.org/10.17504/protocols.io.x9qfr5w) contains detailed protocols.

### 2.6. Transcriptomics Analysis

Transcriptomic analysis was carried out as previously described [36,37]. RNA-Seq reads were quantified using ikra v1.2.2 [38]—an RNA-Seq pipeline centered on Salmon. Trim Galore! v0.6.6 and Cutadapt v3.2 were used to process FASTQ files from transcriptome sequencing using the default parameters [39,40]. Using Salmon v1.4.0 and default parameters, trim FASTQ files were quantified in transcripts per million (TPM) [41]. In order to analyze Gene Ontology (GO) data from RNA-Seq, count tables were imported into integrated differential expression and pathway analysis (iDEP) v 0.91 [42]. At least one sample of quantified transcript readings was filtered at 0.5 counts per million (CPM) and log2-transformed (CPM + c) using EdgeR with a pseudocount of 4. iDEP’s gene set enrichment analysis was carried out using the DESeq2 fold-change values. False-positive rates with q < 0.05 were deemed enriched and subjected to further research. The TRRUST method [43,44], which is used to analyze human transcriptional regulatory interactions, was applied using the web-based Metascape algorithm [45].

### 2.7. Wound Healing Assay

Cells were seeded in 60 mm dishes and cultured overnight. We used a sterile pipette tip to scratch confluent monolayer cells, which were then cultured in the absence or presence of 500 ng/mL TNFα with 100 ng/mL IL-1β and exposed for 24 h to 20% or 1% O_2_ and 5% CO_2_ balanced with nitrogen. Images for each scratch were recorded at 0, 6, 12, 18, and 24 h using a BZ-9000 fluorescence microscope (Keyence, Itasca, IL, USA). Quantification was performed by measuring the number of pixels in each wound closure area using Image J v1.51 (National Institutes of Health, Bethesda, MD, USA).

### 2.8. Statistical Analysis

Data are presented as the mean ± standard deviation (SD). Differences between groups were evaluated using one-way analysis of variance (ANOVA), followed by Dunnett’s or Tukey’s test for multiple comparisons. GraphPad Prism 8 was used to conduct the statistical analysis (GraphPad Software, La Jolla, CA, USA). The cutoff for statistical significance was set to *p* < 0.05.

## 3. Results

### 3.1. Pro-Inflammatory Agents Induce HIF-1α Protein Accumulation under Non-Hypoxic Conditions in Endometrial Epithelial Cells

**Figure 1:**
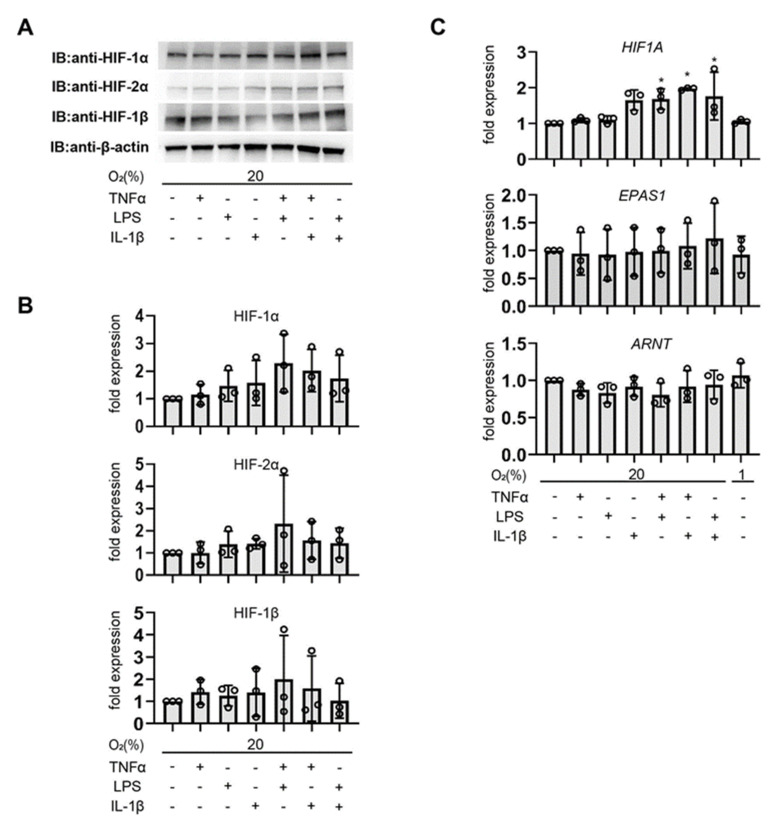
Under non-hypoxic situations, pro-inflammatory substances tend to cause the buildup of HIF-1 protein in endometrial epithelial cells. Immortalized endometrial epithelial cells EM-E6/E7/TERT were incubated with culture media (-), TNFα (500 ng/mL), IL-1β (100 ng/mL), and LPS (500 ng/mL) under 20% O_2_ conditions for 6 h before protein and total RNA were extracted. (**A**) Illustrations of immunoblots. Immunoblotting was performed on whole-cell lysates for HIF-1α, HIF-2α, HIF-1β, and β-actin. There were at least three such experiments carried out. (**B**) Densitometric analysis results that have been β-actin-normalized. (**C**) After normalizing the results to the total RNA levels of EF-1α, semi-quantitative RT-PCR was used to examine the total RNA for the presence of *HIF1A*, *EPAS1*, and *ARNT*. Data are presented as the mean (*n* = 3) and standard deviation (SD). * *p* < 0.05 in comparison to the control group (20% O_2_ circumstances without cytokine therapy).

To investigate the effects of pro-inflammatory cytokines and LPS on the activation of transcription factor HIFs, including HIF-1 and HIF-2, the immortalized endometrial epithelial cells EM-E6/E7/TERT were exposed to TNFα, IL-1β, LPS, or DMOG under 1% or 20% O_2_ for 6 h (Figure 1A and Figure S1). HIF-1α and HIF-2α protein accumulation was induced by exposure to 1% O_2_ or DMOG (1 mmol/L) under 20% O_2_ conditions (Figure S1). An atmosphere of 1% O_2_ and DMOG are authoritative stimuli for HIF-1 activation in endometrial epithelial cells as well. Exposure to TNFα (500 ng/mL), IL-1β (100 ng/mL), or LPS (500 ng/mL) alone did not alter the protein expression of HIF-1α under normoxia. Although not statistically significant in the densitometric evaluation, we observed a reproducible induction of HIF-1α protein in response to combined exposure to these cytokines, even under normoxic conditions. HIF-2α and HIF-1β protein levels were unaffected by any treatment (Figure 1B). We examined the expression of HIF-1α, HIF-2α, and HIF-1β mRNAs using semi-quantitative qRT-PCR and found that TNFα, IL-1β, and LPS alone did not affect HIF-1α mRNA levels, but in combination, they increased HIF-1α mRNA levels within 6 h. Exposure to 1% O_2_ did not alter HIF-1α mRNA levels. The mRNA levels of HIF-2α and HIF-1β were not affected by inflammatory cytokines, LPS, or hypoxia (Figure 1C).

**Figure 2:**
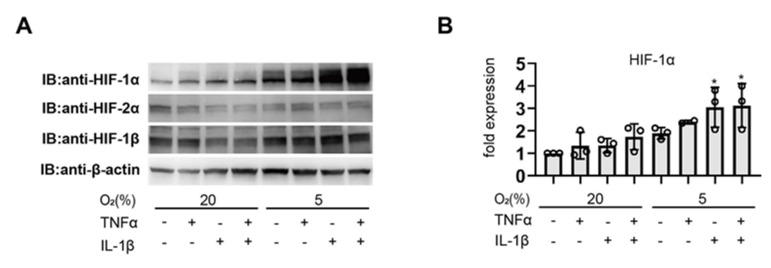
Inflammatory cytokines and hypoxia synergistically increase HIF-1α protein accumulation. (**A**,**B**) Representative immunoblots. EM-E6/E7/TERT cells were exposed to culture media (–), TNFα (500 ng/mL), and IL-1β (100 ng/mL) under 20% or 5% O_2_ conditions for 6 h. Whole-cell lysates were immunoblotted for HIF-1α, HIF-2α, HIF-1β, and β-actin following treatment. There were at least three such experiments carried out. (B) Densitometric analysis results that have been β-actin-normalized. The data are presented as mean SD (*n* = 3). * *p* < 0.05 in comparison to the control group (20% O_2_ circumstances without cytokine therapy).

Next, we examined HIF activation under 5% O_2_ conditions, which is close to the O_2_ environment in the uterus. IL-1β or TNFα and IL-1β treatment enhanced HIF-1α protein expression induced by 5% oxygen conditions (Figure 2A,B). Pro-inflammatory agents and hypoxic stimuli synergistically induced HIF activation. 

### 3.2. Involvement of the PI3K and MAPK Signaling Pathway in Cytokine-Induced HIF-1 Activation

**Figure 3:**
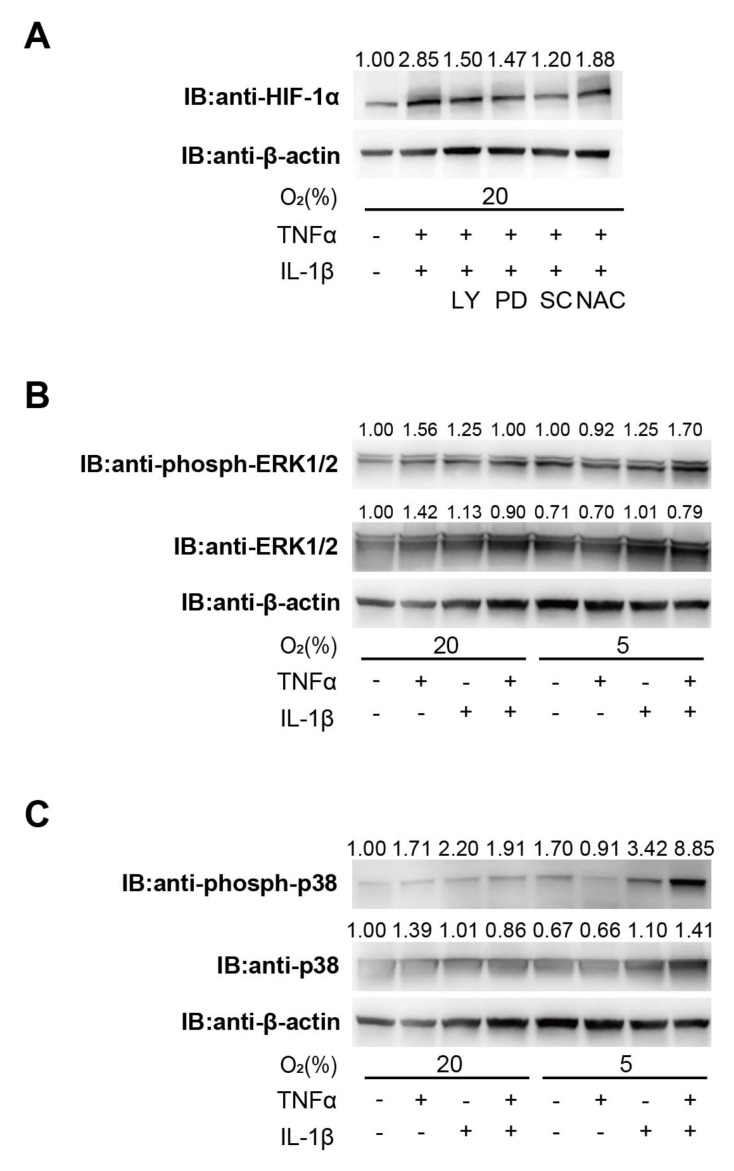
Involvement of PI3K and MAPK signaling pathways in cytokine-induced HIF-1 activation. (**A**) EM-E6/E7/TERT cells were exposed to TNFα (500 ng/mL) and IL-1β (100 ng/mL) with or without the indicated kinase inhibitors (25 µmol/L LY, 20 µmol/L PD, and 100 µmol/L SC) or 10 mM N-acetyl-L-cysteine (NAC) for 6 h under 20% O_2_ conditions, and whole-cell lysates were immunoblotted for HIF-1α and β-actin. LY—LY294002; PD—PD98059; SC—SC-514. (**B**,**C**) EM-E6/E7/TERT cells were exposed to culture media (–), TNFα (500 ng/mL), and IL-1β (100 ng/mL) under 20% or 5% O_2_ conditions for 6 h. After the treatment, whole-cell lysates were immunoblotted for phospho-ERK1/2, ERK1/2 (**B**), phospho-p38, p38 (**C**), and β-actin. (**A**–**C**) The densitometric analysis’ findings, normalized by -βactin, are displayed as the values above the immunoblots.

It is known that the PI3k-Akt-mTOR pathway promotes HIF-1α translation, and in parallel with this pathway, a MAPK-mediated increase in HIF-1α translation has also been reported [46]. We investigated the signaling pathway involved in pro-inflammatory cytokine-induced HIF activation in EM-E6/E7/TERT cells. We investigated the effects of kinase inhibitors to determine whether PI3K or MAPK signaling was involved in the regulation of HIF-1 protein levels. We discovered that LY294002 (25 µmol/L), PD98059 (20 µmol/L), and SC-514 (100 µmol/L) attenuated TNFα- (500 ng/mL) and IL-1β (500 ng/mL)-induced HIF-1α protein expression (Figure 3A). We also investigated the phosphorylation of ERK1/2 and p38 proteins exposed to pro-inflammatory cytokines or hypoxia in EM-E6/E7/TERT cells. Treatment with TNFα and IL-1β or hypoxia increased the levels of phosphorylated p38 and ERK. We detected further phosphorylation of ERK1/2 and p38 as the levels of inflammatory cytokines increased during hypoxia. ERK1/2 and p38 protein expression was not affected by cytokine or hypoxic treatment (Figure 3B,C).

### 3.3. Global Gene Expression Analysis Using RNA-Seq

**Figure 4:**
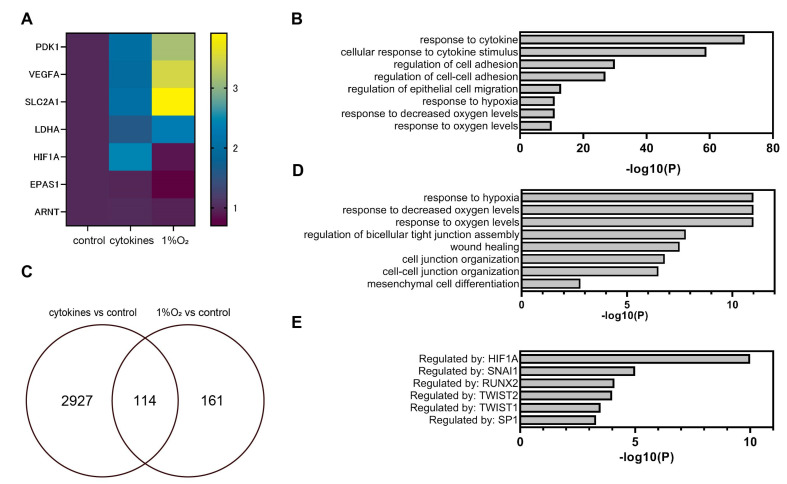
Global gene-expression analysis using RNA-Seq and next-generation sequencing (NGS). (**A**–**E**) EM-E6/E7/TERT cells were exposed to 1% O_2_ or pro-inflammatory cytokines (500 ng/mL TNFα and 100 ng/mL IL-1β) under 20% O_2_ conditions for 8 h, and total RNAs were analyzed by RNA-Seq. (**A**) Expression of genes regulated by HIF-1 and the expression of *HIF1A*, *EPAS1*, and *ARNT*. (**B**) GSEA, with the gene sets whose expression ratios were significantly altered under pro-inflammatory cytokine exposure separated according to the gene ontology (GO) term. (**C**) Genes with significantly altered expression. (**D**) GSEA, with the gene sets whose expression was commonly upregulated by pro-inflammatory cytokine treatment and hypoxia exposure separated according to GO term. (**E**) Analysis using the TRRUST database of pathways commonly enriched by pro-inflammatory cytokine treatment and hypoxia exposure.

To investigate the effects of proinflammatory cytokines and hypoxia (1% O_2_ conditions), we used RNA-Seq to examine the patterns of gene expression throughout the entire genome. EM-E6/E7/TERT cells were exposed to 1% O_2_ or either TNF (500 ng/mL) or IL-1 (100 ng/mL) for 8 h (Table S1). For differential expression and pathway analysis, the expression matrix, which represents transcripts per kilobase million (TPM) values aggregated at the gene level, was utilized. The results of the principal component analysis (PCA) and hierarchical clustering analysis are presented in Figure S2. Among the genes whose expression was regulated by transcription factor HIF-1, the expression of *PDK1*, *VEGFA*, *SLCA2A1*, and *LDHA* was demonstrated as a heatmap with *HIF1A* and *EPAS1*. The expression of *PDK1*, *VEGFA*, *SLCA2A1*, and *LDHA* was induced by cytokine or hypoxic treatment (Figure 4A). Interestingly, and consistent with our qRT-PCR results (Figure 1C), *HIF1A* expression was induced by the cytokine treatment. Gene set enrichment analysis (GSEA) was used to compare genes whose expression ratios were significantly altered under pro-inflammatory cytokine exposure (Figure S3) and hypoxic conditions (Figure S4). We observed the enrichment of some pathways associated with inflammation (Figure 4B and Figure S3, Table S2). In addition to the inflammatory pathways, the “response to hypoxia” (GO:0001666) pathway was identified with statistical significance (LogP < –11). “Responses to decreased oxygen levels” (GO:0036293) and “response to oxygen levels” (GO:0070482) were also identified as enriched pathways. Figure 4B displays the expression levels of the selected genes inside GO:0071456 (cellular response to hypoxia). Pathways such as wound healing (GO:0042060) and epithelial cell migration (GO:0010631) were also enriched. From a list of common genes (those upregulated by cytokine treatment or hypoxic exposure; Figure 4C and Figure S5, Table S3), we identified GO pathways related to oxygen levels in the GSEA. GO:0048762 (mesenchymal cell differentiation) was also identified (Figure 4D). In addition, pathways related to tight junction formation, wound healing, and focal adhesions were enriched. Analysis using the TRRUST database made it abundantly evident that the enriched pathways were controlled by the gene products of the *HIF1A*, *SNAI1*, *RUNX2*, *TWIST1*, *TWIST2*, and *SP1* genes (Figure 4E). Thus, treatment with cytokines activated signaling pathways similar to those activated by hypoxic exposure. The enriched pathways were regulated by HIFs and EMT-related transcription factors, according to analysis using the TRRUST database.

### 3.4. Representative Genes of EMT were Induced by Cytokines

**Figure 5:**
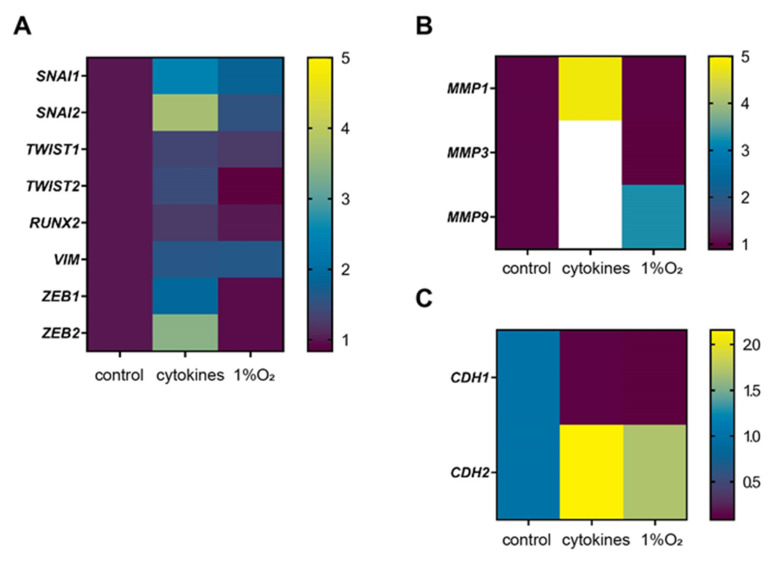
Representative genes of EMT induced by cytokines. Genes for transcription factors associated with (**A**) EMT, (**B**) MMP, (**C**) and cadherin, identified using the TRRUST database.

Further analysis by GSEA revealed genes encoding transcription factors associated with the EMT, including *SNAI1*, *TWIST1*, *TWIST2*, *RUNX2*, *ZEB1*, *ZEB2*, *MMP1*, *MMP3*, and *MMP9* (Figure 5A,B). Notably, cytokine stimulation decreased the expression of *CDH1* (cadherin-1, E-cadherin) and increased the expression of *CDH2* (cadherin-2/N-cadherin), indicating that cytokine stimulation induced a so-called “cadherin switch” (Figure 5C). We attempted to detect this gene expression change at the protein level. However, we were unable to show the cadherin switch at the protein level due to poor detection with several specific antibodies.

### 3.5. Cytokines Facilitate Morphological Changes in EM-E6/E7/TERT Cells

**Figure 6:**
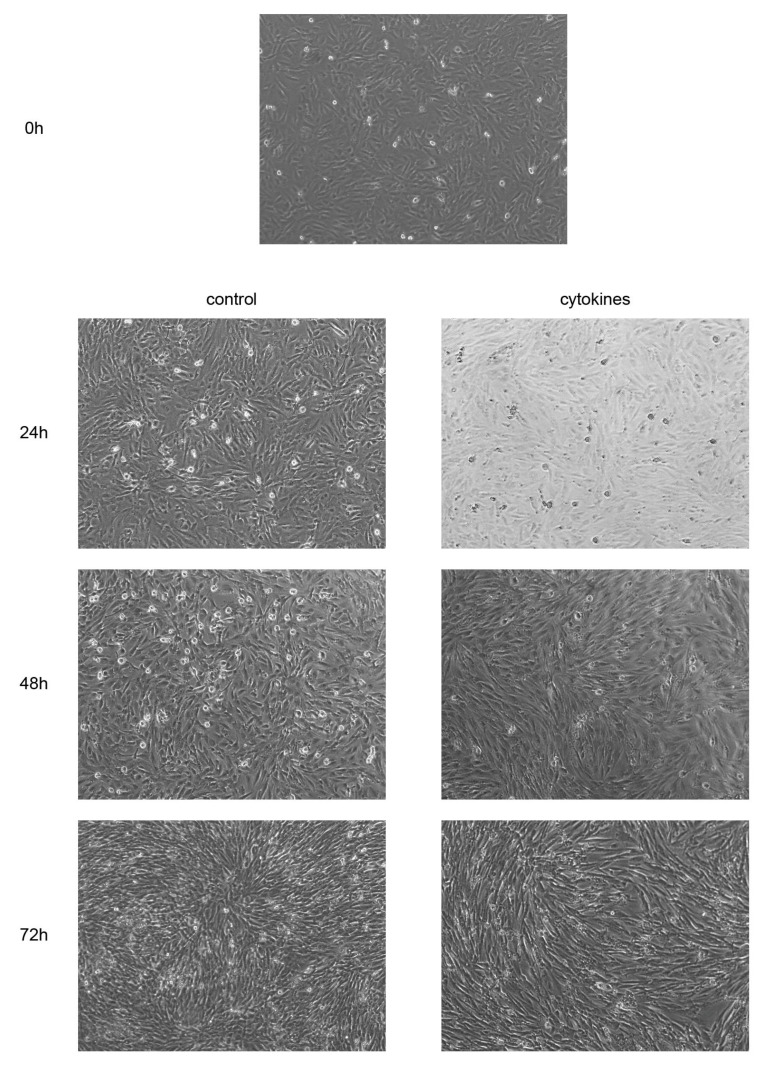
Effects of pro-inflammatory cytokines on cell morphology. EM-E6/E7/TERT cells were cultured with or without TNFα (500 ng/mL) and IL-1β (100 ng/mL) for 72 h. Original magnification ×100.

We examined the effects of cytokine stimulation on cell phenotypes. EM-E6/E7/TERT cells were cultured for 24 h, treated with 500 ng/mL of TNFα and 100 ng/mL of IL-1β, and observed over 72 h. When cultured in the presence of pro-inflammatory cytokines, the cells changed from polygon- to spindle-shaped, became more flattened, and spread extensively. Morphological changes were visible from 24 h after the treatment and progressed gradually until 72 h (Figure 6).

### 3.6. Cytokines Facilitate Wound Healing

**Figure 7:**
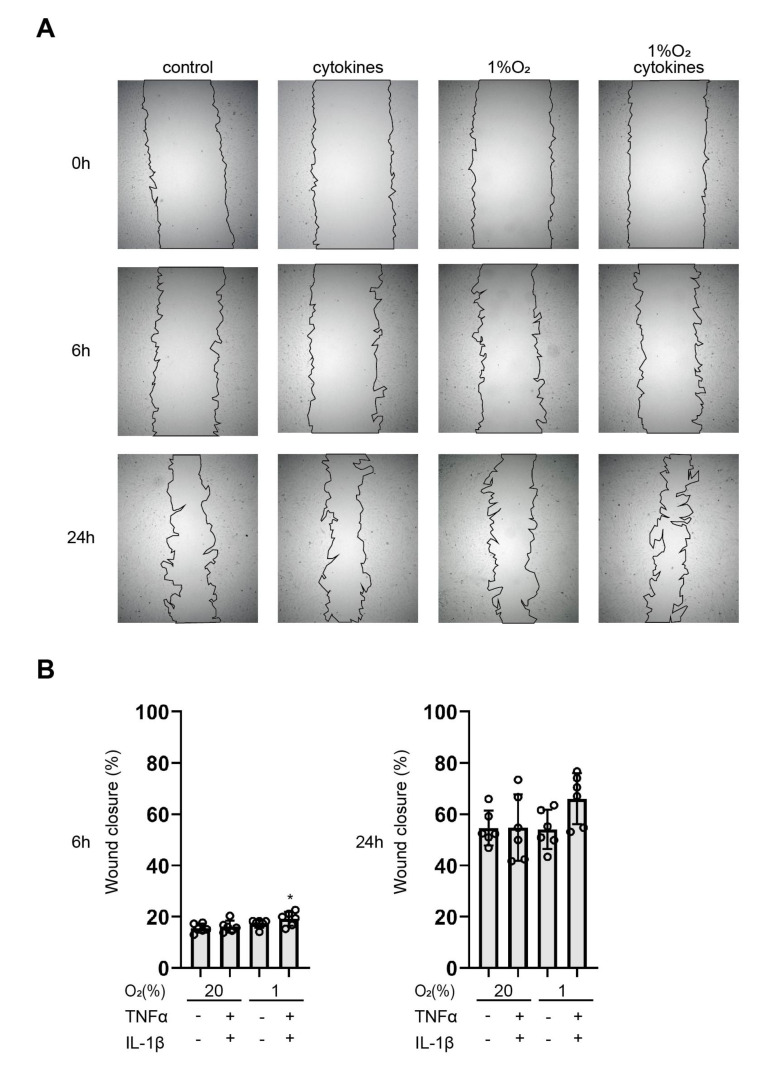
Cytokines facilitate wound healing. Confluent monolayers of EM-E6/E7/TERT cells were wounded with a uniform scratch, washed to remove cell debris, and cultured for 24 h under 1% or 20% O_2_ conditions in the absence or presence of TNFα (500 ng/mL) and IL-1β (100 ng/mL). (**A**) Phase contrast images of cell cultures after 0 h (upper 4 panels), 6 h (middle 4 panels), and 24 h (lower 4 panels) are presented. (**B**) Measurements of colonized areas. “Percentage wound closure” was defined as: (area at time 0—area at each time/area at time 0) × 100. Data represent the mean ± SD (*n* = 6). * *p* < 0.05 compared to the control.

A strong migratory potential is one of the most significant features of the mesenchymal state. We performed a wound-healing assay to examine the effects of pro-inflammatory cytokines and hypoxia on cell migration. Confluent monolayers of EM-E6/E7/TERT cells were scratched to form wounds and cultured under 1% or 20% O_2_ conditions in the absence or presence of TNFα (500 ng/mL) and IL-1β (100 ng/mL, Figure 7A). Proinflammatory cytokines under hypoxia augmented wound healing, and the effect was significant after 6 h (Figure 7B). In contrast, treatment with pro-inflammatory cytokines or hypoxia alone did not upregulate the migration ability of EM-E6/E7/TERT cells.

### 3.7. Effect of HIF-1 Suppression by Echinomycin on EMT-Related Genes

**Figure 8:**
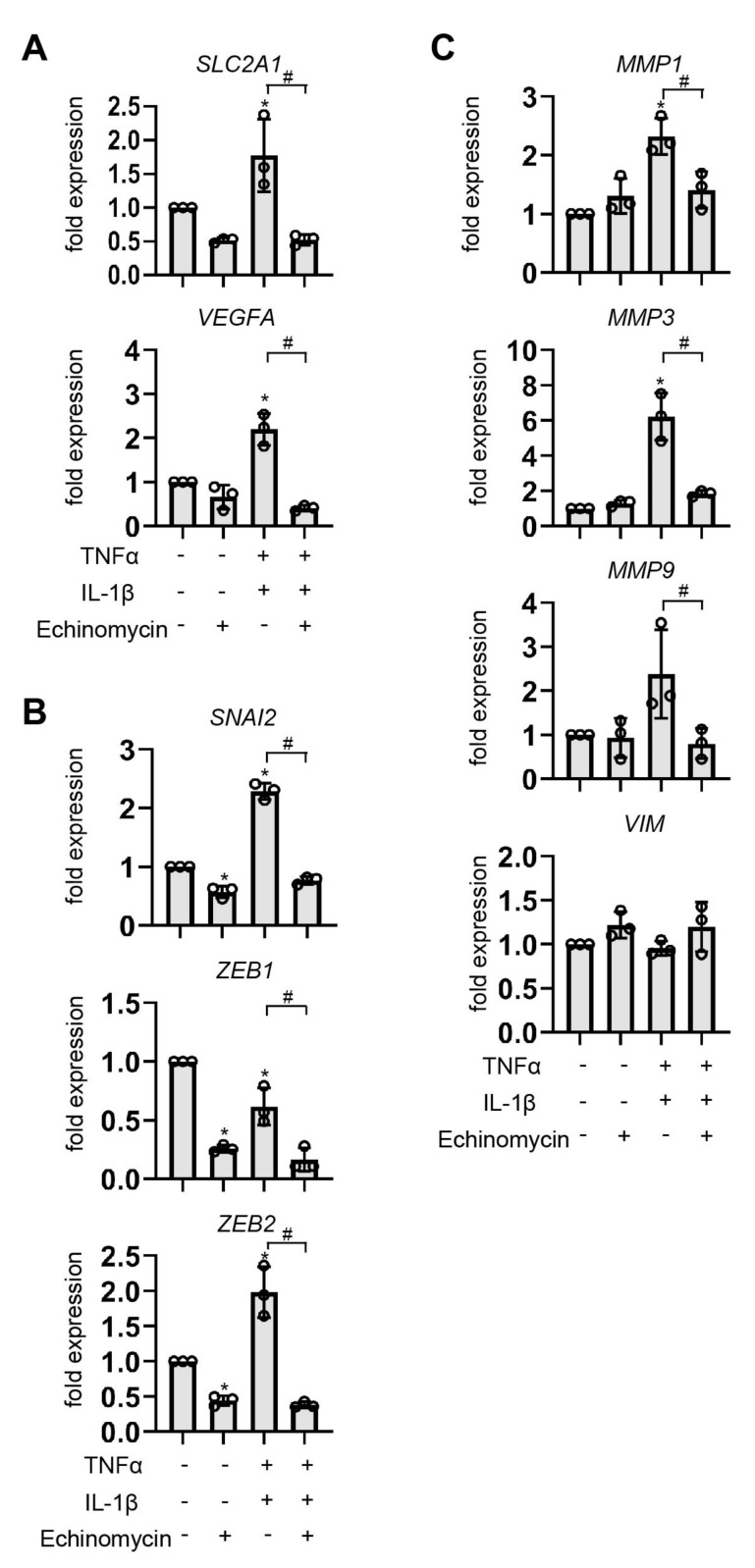
Effect of HIF-1 suppression by echinomycin on EMT-related genes. (**A**–**C**) EM-E6/E7/TERT cells were cultured under 20% O_2_ conditions for 8 h in medium containing either control or pro-inflammatory cytokines (500 ng/mL TNFα and 100 ng/mL IL-1β) and/or echinomycin (10 nmol/L). Total RNAs were analyzed by semi-quantitative RT-PCR and calculated after normalization to EF-1α total RNA levels. Data represent the mean ± SD (*n* = 3). * *p* < 0.05 compared to the control. # *p* < 0.05 for the indicated comparison.

We used echinomycin—a small-molecule inhibitor of HIF-1 activity—to determine whether HIF-1 is mediated in the EMT induced by pro-inflammatory cytokines. Treatment with 10 nM of echinomycin inhibited cytokine-induced *VEGFA* and *SLC2A1* expression (Figure 8A). We examined the effect of echinomycin on the mRNA levels of *SNAI2*, *ZEB1*, *ZEB2*, *VIM*, *MMP1*, *MMP3*, and *MMP9* with or without the addition of echinomycin in EM-E6/E7/TERT cells and found that the expression levels of *SNAI2* and *ZEB2* mRNA were significantly increased by the pro-inflammatory cytokine treatment and suppressed by echinomycin. Even in the controls, the echinomycin treatment inhibited *SNAI2* and *ZEB2* mRNA expression levels. *ZEB1* mRNA levels were not increased by pro-inflammatory cytokines but were suppressed by echinomycin (Figure 8B). The mRNA expression of *MMP1*, *MMP3*, and *MMP9* mRNA was upregulated by the treatment with inflammatory cytokines (Figure 8C).

## 4. Discussion

We showed that pro-inflammatory factors including TNFα, IL-1β, and LPS in concert with hypoxic environments activate HIF-1 and promote the EMT in endometrial epithelial cells. Although it has been reported for immunocompetent cells and cells of other organs, this is the first report of cytokine-induced HIF-1 activation in endometrial epithelial cells. The human endometrium has a physical and immunological barrier in the form of a glandular epithelial cell layer. The penetration of this glandular epithelial barrier is essential for the embryo to attach to the endometrial stroma during human implantation. The destruction of the epithelial barrier by the embryo must be rapidly repaired to protect the uterus and embryo from infection and avoid implantation failure. The EMT is a phenomenon in which epithelial cells acquire the traits of mesenchymal cells in a coordinated and continuous biological response with a stepwise mechanism. It can be viewed as a combination of essential functions, such as cell adhesion, proliferation, differentiation, and motility. The invasion of embryos into the endometrium is thought to require EMT-associated changes in the endometrial epithelium, a barrier at the time of implantation, and evidence supports the presence of the EMT in the endometrial epithelium [47].

In this study, we demonstrated that inflammatory cytokine stimulation induced HIF-1 activation and the EMT in EM-E6/E7/TERT cells. Hypoxia is common on the surface of the human endometrium; during early human pregnancy, the oxygen concentration at the endometrial surface is 18 mmHg (2.5% O_2_ conditions) and that within the endometrium is 40 mmHg (about 5% O_2_ conditions) [48,49]. Furthermore, the endometrial luminal epithelium is distant from any blood vessel and separated from the endometrial stroma by a basement membrane. Matsumoto et al., in a study incorporating transgenic mice, found that HIFs regulate the process of embryonic invasion in the endometrium and HIF-2α promotes the shedding of the luminal epithelium to expose the endometrial stroma, ultimately allowing for the implantation of the embryo in the uterus [50]. Consistent with previous reports, we showed that a decrease in the oxygen partial pressure in endometrial epithelial cells led to the intracellular accumulation of both HIF-1α and HIF-2α proteins. Since its molecular cloning in 1995 [51], the oxygen partial pressure-dependent regulation of HIF has been comprehensively described. However, the partial pressure-independent regulation of HIF activation has also been reported. In cells lacking or mutated in VHL, as is often the case in renal carcinoma, HIF activation is observed even in the absence of hypoxia [52]. Similarly, deletions or mutations in tumor-suppressor genes, such as *TP53* and *PTEN*, may also result in HIF activation [7,31]. Signals from growth factors such as epidermal growth factor (EGF), insulin-like growth factor (IGF)-1, orexin, muscarinic receptors, and nicotinic receptors mediated by acetylcholine and nicotine also result in HIF-1 activation [8,53,54]. In the present study, inflammatory cytokine treatment increased HIF-1α but not HIF-2α mRNA expression in EM-E6/E7/TERT cells; interestingly, hypoxic stimulation did not affect HIF-1α mRNA expression. Several studies have shown that inflammatory cytokines induce HIF-1α mRNA expression by activating NF-κB [55,56]. MAPK and NF-κB may also be involved in a reactive oxygen species (ROS)-dependent manner. The ROS scavenger, NAC, inhibited the induction of HIF-1α protein by cytokines. We found that treatment with LY294002 and SC-514 suppressed cytokine-mediated HIF-1α levels, which is consistent with earlier publications showing that the activation of the PI3K/Akt/mTOR and MAPK pathways increased the rate of HIF-1α mRNA expression and protein synthesis [46,57,58,59]. The induction of HIF-1α protein expression by inflammatory cytokines was blocked by PI3K or MAPK inhibition (Figure 3A). These cytokines induced the phosphorylation of p42/44 MAPK and p38 MAPK, both of which are involved in protein translation. Collectively, our findings indicate that cytokines increase the rate of HIF-1α protein synthesis by increasing HIF-1α mRNA levels and activating the PI3K/Akt and MAPK pathways.

The RNA-Seq analysis followed by GSEA clearly indicate that cytokines’ stimulation activates cytokine and hypoxia-related signaling pathways as well as the pathways related to the EMT. Of the common genes induced by cytokines and hypoxia, genes related to the HIF-1 pathway, the EMT, wound healing, and tight junction assembly were also induced. When common genes were analyzed against the TRRUST database, transcription factors closely related to EMT, such as *HIF1A*, *SNAl1*, *TWIST2*, *TWIST1*, and *SP1,* were also enriched. Cytokine stimulation resulted in the elevated expression of EMT markers and the increased activation of EMT-related transcription factors, such as *SNAI1* and *ZEB1*. In addition, the expression of *MMP2* and *MMP9* and the motility of endometriotic epithelial cells were upregulated. Thus, EMT-related pathways are activated in endometrial epithelial cells exposed to a high-cytokine and low-oxygen-partial-pressure environment. These results clearly illustrated the involvement of the HIF-1 pathway in promoting EMT-associated phenotypic changes in endometrial epithelial cells.

Endometritis is a chronic inflammatory disease, and immune and inflammatory responses play an important role in its pathogenesis, including inflammatory cytokines, chemokines, proteases, prostaglandins, hormone receptors, and angiogenic factors. In the current study, we found that endometrial epithelial cells were activated by hypoxic exposure and the inflammatory cytokine stimulation of HIF. We also showed a synergistic effect between hypoxic exposure and inflammatory cytokine stimulation. Epithelial cell layers are generally formed by homodimers of the intercellular adhesion molecule E-cadherin, which firmly bind to each other. The cell–cell adhesion of mesothelial-like cells, which retain high motility, is also mediated by E-cadherin, but more frequently by N-cadherins. In the EMT, molecules responsible for cell–cell adhesion switch their main roles from E-cadherin to N-cadherin. This cadherin switch also occurred in the present study based on the changes in the expression of the related genes.

We acknowledge some limitations of our study design. Gene expression profiling using RNA-seq followed by qPCR validation indicated the close link between the hypoxic response and tissue remodeling, which remains to be investigated at the protein level. Further studies are required to explore the mechanisms underlying the interaction between the HIF-1 pathway and the EMT of endometrial epithelial cells. A detailed understanding of the temporal and spatial regulation of HIF-1 activity in endometritis will also await the generation of a suitable experimental system. Another limitation is that we used an immortalized cell line instead of primary-cultured cells. Few related studies have used primary-cultured cells since endometrial epithelial cells are difficult to isolate and cannot tolerate long-term culture. Additionally, due to individual variation, results are often inconsistent in studies employing primary-cultured endometrial epithelial cells. Our in vitro study design allows for the quantitative analysis of fundamental cellular processes, such as adhesion and motility, without relying solely on qualitative observations, which represents a significant improvement over in vivo studies, even in human research, which is subject to many ethical and social restrictions. Nevertheless, our findings require validation by in vivo studies.

## 5. Conclusions

**Figure 9:**
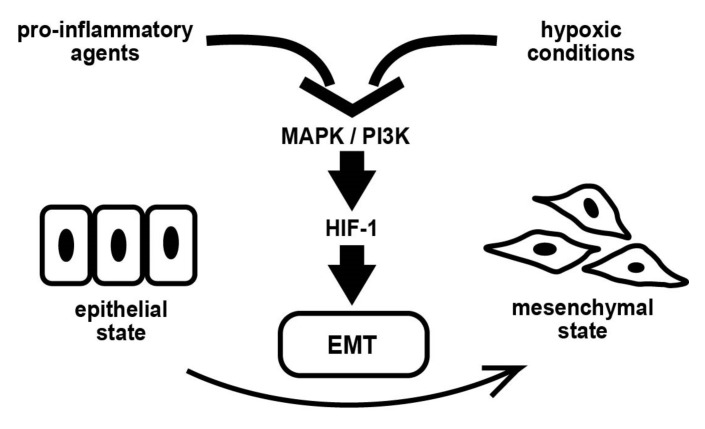
Overall scheme of the effects of pro-inflammatory agents in endometrial epithelial cells. Abbreviations: HIF-1—hypoxia-inducible factor-1; EMT—epithelial–mesenchymal transition.

We used comprehensive gene expression analysis to show that EMT-related pathways are activated by inflammatory cytokine stimulation, and the expression of these molecules is renewed in a hypoxic environment dependent on HIF-1 activation (Figure 9). Our results indicate that the intrauterine oxygen environment and inflammatory state establish crosstalk that drives the EMT in a complex manner.

## Figures and Tables

**Table 1:** Primers used for the real-time polymerase chain reaction (PCR).

Target	Gene Symbol	Forward Primer 5′–3′	Reverse Primer 5′–3′
HIF-1a HIF-2a HIF-1b GLUT1 VEGF SNAI2 ZEB1 ZEB2 MMP1 MMP3 MMP9 Vimentin EF1α	HIF-1A EPAS1 ARNT SLC2A1 VEGFA SNAI2 ZEB1 ZEB2 MMP1 MMP3 MMP9 VIM EEF1A1	ACACACAGAAATGGCCTTGTGA ATGGGACTTACACAGGTGGAG TGTTGGCTACCAGCCACAGGAACT TCCACGAGCATCTTCGAGA CGAAACCATGAACTTTCTGC TGTGACAAGGAATATGTGAGCC TTACACCTTTGCATACAGAACCC CAAGAGGCGCAAACAAGCC ACAAACCCCAAAAGCGTGTG TTCGTTTTCTCCTGCCTGTG ATGCCTGCAACGTGAACATC GACGCCATCAACACCGAGTT TCTGGTTGGAATGGTGACAACATGC	CCTGTGCAGTGCAATACCTTC GCTCTGTGGACATGTCTTTGC ACCGGAACCGGAACATGACAGA ATACTGGAAGCACATGCCC CCTCAGTGGGCACACACTCC TGAGCCCTCAGATTTGACCTG TTTACGATTACACCCAGACTGC GGTTGGCAATACCGTCATCC AGAAGGGATTTGTGCGCATG AGCAGCAGCCCATTTGAATG AGAATCGCCAGTACTTCCCATC CTTTGTCGTTGGTTAGCTGGT AGAGCTTCACTCAAAGCTTCATGG

## Data Availability

The data presented in this study are openly available in the DNA Data Bank of Japan Sequence Read Archive (https://www.ddbj.nig.ac.jp/dra/index-e.html, accessed on 14 July 2022; accession nos. DRR395305–DRR395313).

## Supplementary Materials

The following supporting information can be downloaded at: https://www.mdpi.com/article/10.3390/biomedicines11010210/s1, Figure S1: Immunoblots for HIF-1α, HIF-2α, HIF-1β, and β-actin under hypoxic conditions; Figure S2: Principal component analysis (PCA) for the RNA-Seq results; Figure S3: Results of gene set enrichment analyses upregulated by pro-inflammatory cytokine treatment; Figure S4: Results of gene set enrichment analyses upregulated by exposure to 1% O_2_; Figure S5: Results of gene set enrichment analyses commonly upregulated by pro-inflammatory cytokine treatment and exposure to 1% O_2_; Table S1: The expression matrix, calculated as scaled TPM and loaded at gene level by tximport; Table S2: Gene set enrichment analyses for gene lists using Metascape database between cytokines-treatment and control groups; Table S3: Gene set enrichment analyses for gene lists using Metascape database between 1% O_2_ conditions and control groups.

## Appendix A

**Table A1:** Table of Key Resources.

Reagents	Source	Identifier
12% Mini-PROTEAN^®^ TGXTM Precast Protein Gels	BIO-RAD	4561043
30w/v% Albumin Solution, from Bovine Serum (BSA), Fatty Acid Free	FUJIFILM	017-22231
7.5% Mini-PROTEAN^®^ TGXTM Precast Protein Gels	BIO-RAD	4561023
Anti-rabbit IgG, HRP-linked Antibody	Cell Signaling TECHNOLOGY	7074
Anti-β-Actin antibody	Sigma-Aldrich	A5316
Blocking One	Nacalai Tesque	03953-95
DC^TM^ Protein Assay Reagents Package	BIO-RAD	5000116JA
DMEM, low glucose, pyruvate	Gibco	11885-084
N-(2-methoxy-2-oxoacetyl) glycine methyl ester (DMOG)	Sigma-Aldrich	D3695
Dimethyl Sulfoxide	FUJIFILM	043-07216
Echinomycin	Enzo Life Sciences	ALX-380-201
ECL^TM^ anti-mouse IgG HRP Linked Whole Ab	cytiva	NA931
ECL^TM^ Prime Western Blotting Detection Reagents	cytiva	RPN2232
Fetal Bovine Serum Characterized	Hyclone	SH30396
HIF-1β/ARNT (D28F3) XP^®^ rabbit mAb	Cell Signaling TECHNOLOGY	5537
HIF-2 alpha/EPAS1 Antibody	Novus Biologicals	NB100-122
LY294002	FUJIFILM	1290-4861
Maxwell^®^ RSC simply RNA Cells Kit	Promega	AS1390
N-Acetyl-L-cysteine, ROS inhibitor	abcam	143032
p38 MAPK(D13E1) XP^®^ Rabbit mAb	Cell Signaling TECHNOLOGY	8690
p44/42 MAPK(Erk1/2) (137F5) Rabbit mAb	Cell Signaling TECHNOLOGY	4695
PBS, pH 7.4	Gibco	10010-023
PD98059	FUJIFILM	169-19211
Penicillin-Streptomycin Mixed Solution (Stabilized)	Nacalai Tesque	09367-34
Phospho-p38MAPK(Thr180/Tyr182) (D3F9) XP^®^ Rabbit mAb	Cell Signaling TECHNOLOGY	4511
Phospho-p44/42MAPK(Erk1/2) (Thr202/Tyr204) (D13.14.4E) XP^®^ Rabbit mAb	Cell Signaling TECHNOLOGY	4370
Protease inhibitor cocktail	Calbiochem	539134
Purified Mouse Anti-Human HIF-1α	BD Transduction Laboratories^TM^	610959
Recombinant Human IL-1β	PeproTech	200-01B
Recombinant Human TNF-α	PeproTech	300-01A
ReverTra Ace^®^ qPCR RT Master Mix with gDNA Remover	TOYOBO	FSQ-301
RIPA Buffer	FUJIFILM	182-02451
Running Buffer Solution for SDS-PAGE	Nacalai Tesque	30329-61
Sample Buffer Solution with Reducing Reagent (6×) for SDS-PAGE	Nacalai Tesque	09449-14
SC-514	Sigma-Aldrich	SML0557
THUNDERBIRD^®^ Next SYBR qPCR Mix	TOYOBO	QPX-201
Trans-Blot^®^ Turbo™ Mini PVDF Transfer Packs	BIO-RAD	1704156
Tris Bufferd Saline with Tween^®^ 20 (TBS-T) Tablets, pH7.6	TaKaRa	T9142
Trypsin-EDTA (0.25%)	Gibco	25200-056
Ultrapure LPS, E. coli 0111: B4	InvivoGen	tlrl-3pelps
