# Supplementary material for: Inflammatory Cytokine-Induced HIF-1 Activation Promotes Epithelial–Mesenchymal Transition in Endometrial Epithelial Cells
Source: Biomedicines. 2023 Jan 14;11(1):210. doi: 10.3390/biomedicines11010210 (PMC9855875; doi:10.3390/biomedicines11010210)
Supplement: Supplementary file 1 [file biomedicines-11-00210-s001.zip › Biomedicins_WB_revise.pptx]

## Slide 1
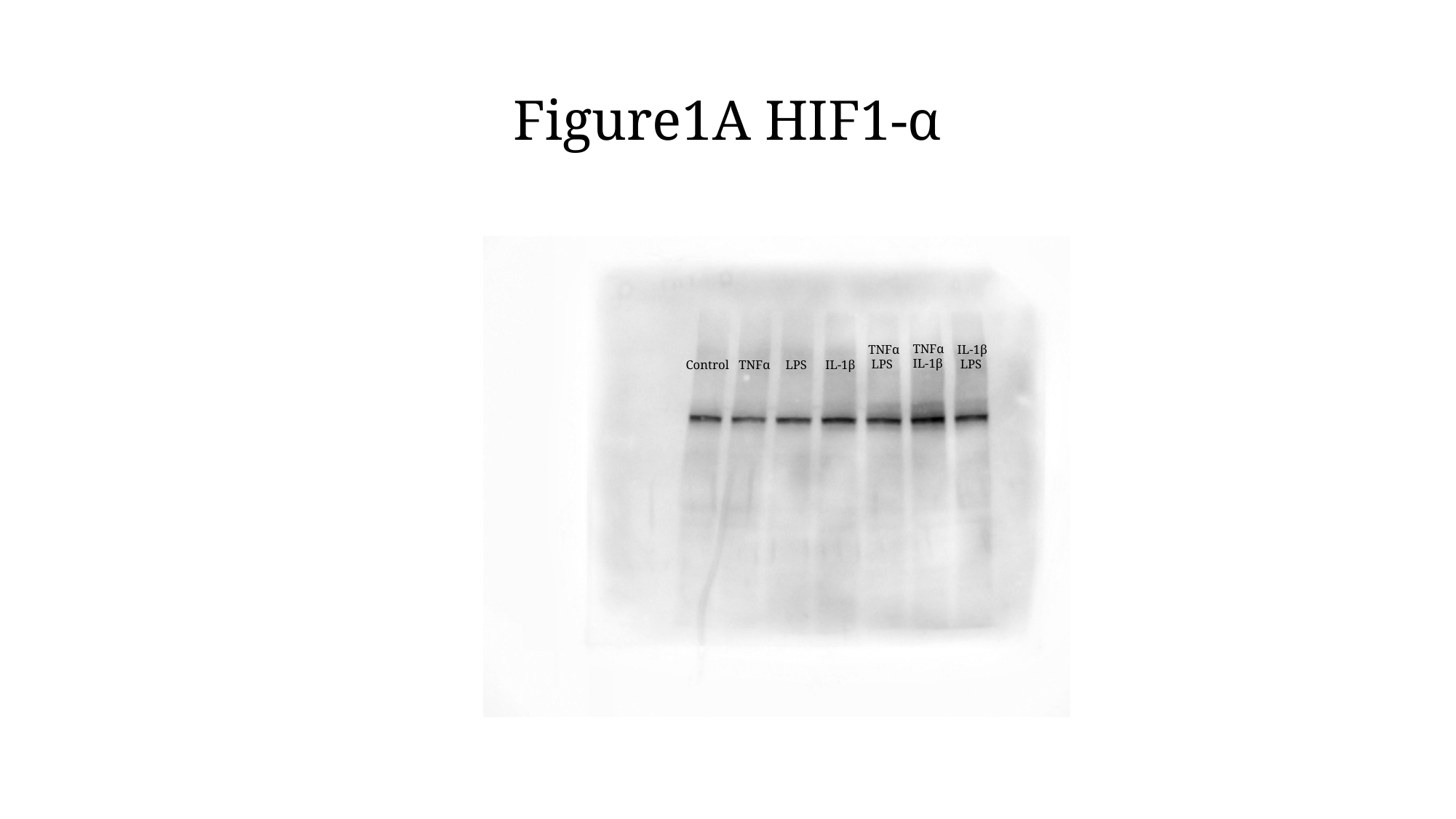

# Figure1A HIF1-α
TNFα
IL-1β
IL-1β
 LPS
TNFα
 LPS
Control
TNFα
LPS
IL-1β

## Slide 2
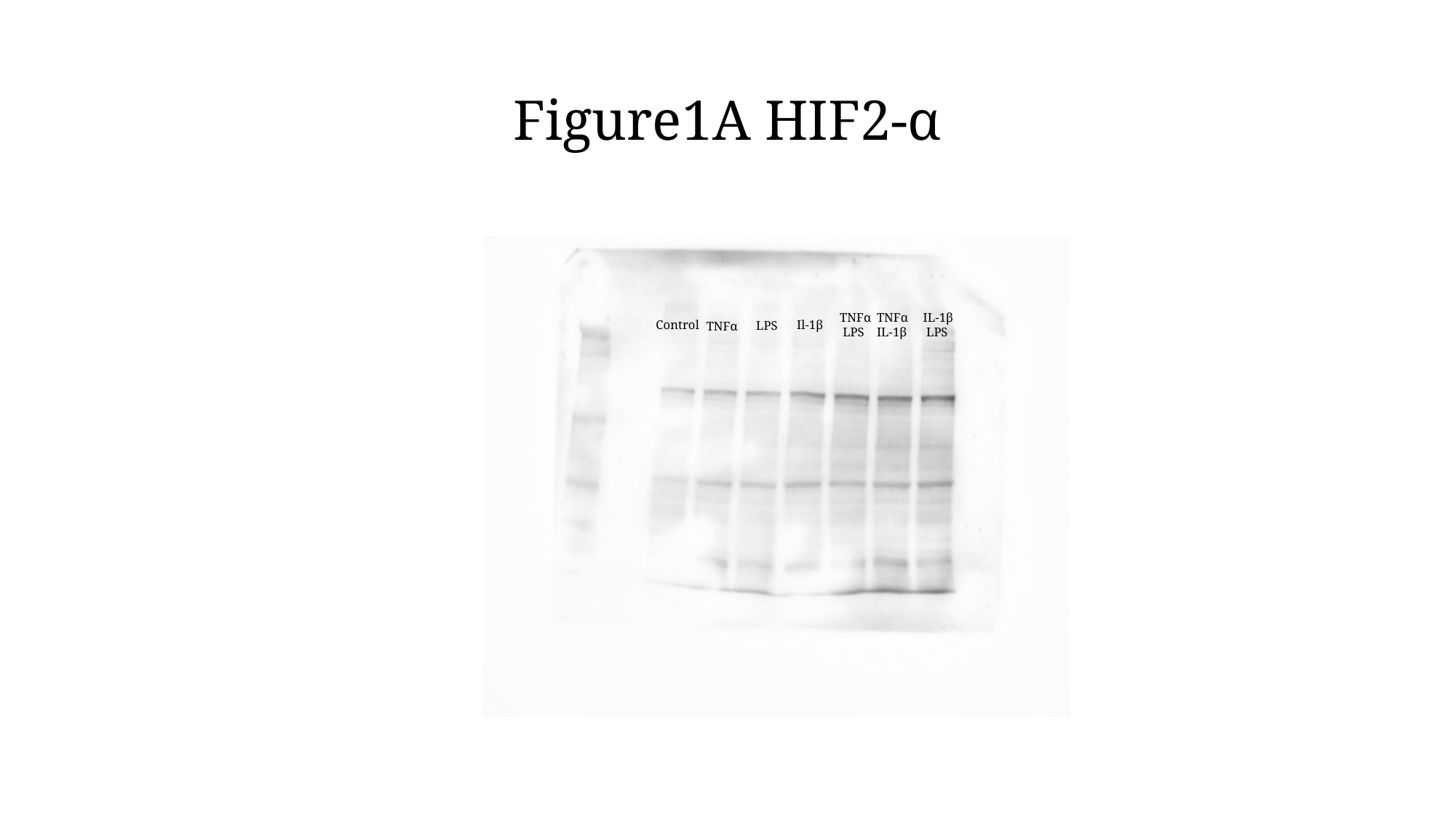

# Figure1A HIF2-α
TNFα
 LPS
TNFα
IL-1β
IL-1β
 LPS
Control
Il-1β
LPS
TNFα

## Slide 3
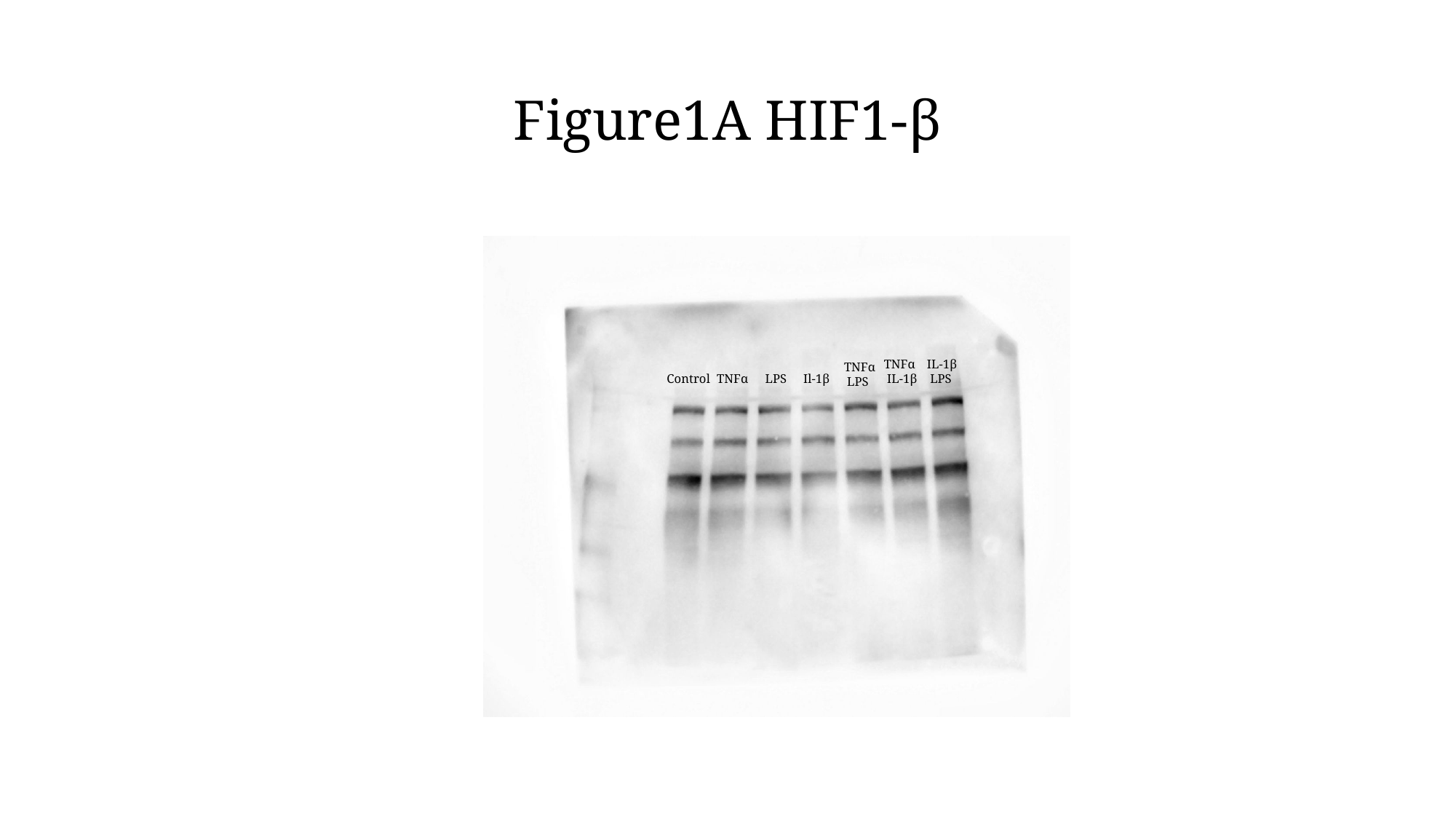

# Figure1A HIF1-β
TNFα
 IL-1β
IL-1β
 LPS
TNFα
 LPS
Control
TNFα
LPS
Il-1β

## Slide 4
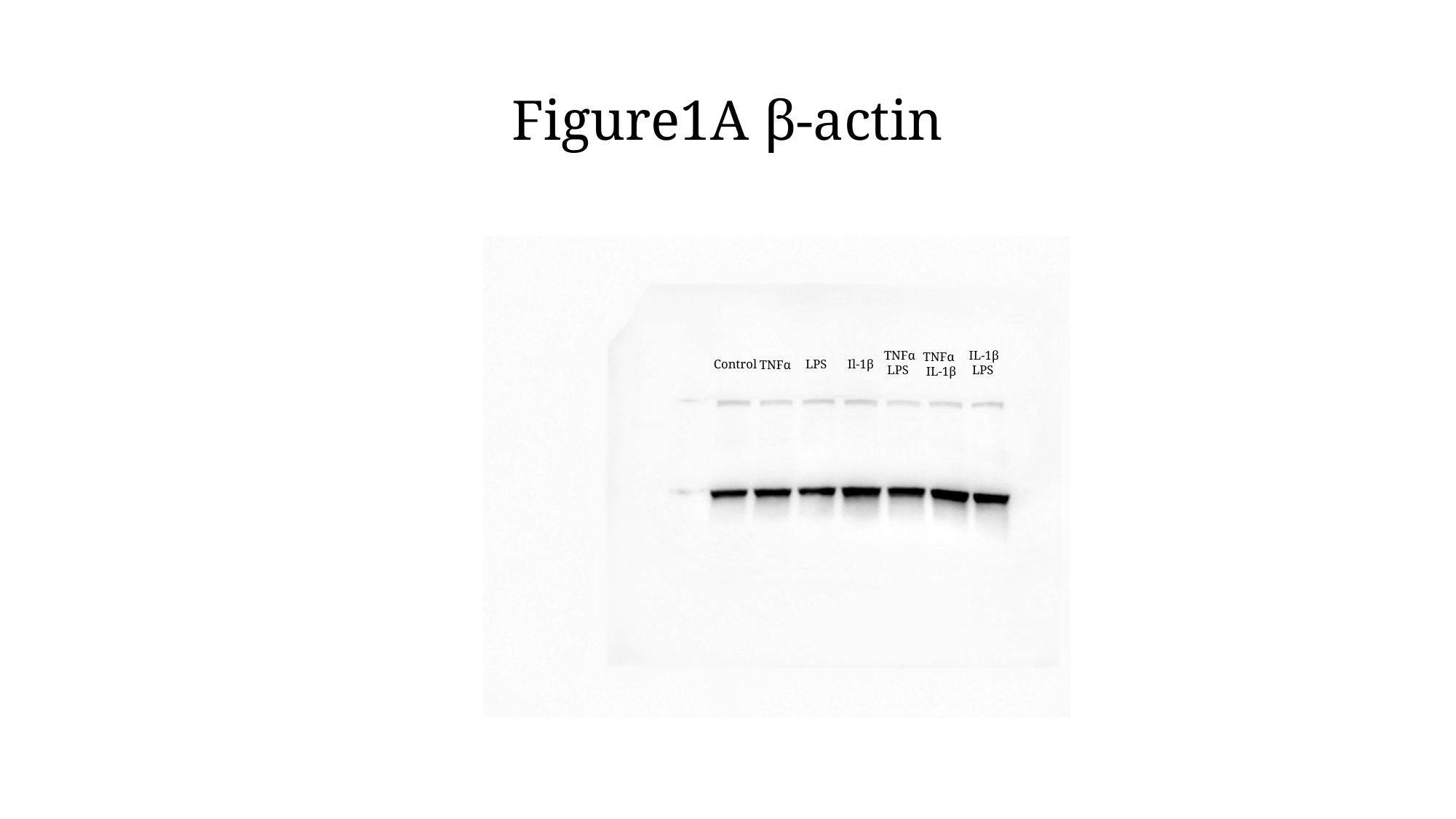

# Figure1A β-actin
TNFα
 LPS
IL-1β
 LPS
TNFα
 IL-1β
Il-1β
LPS
Control
TNFα

## Slide 5
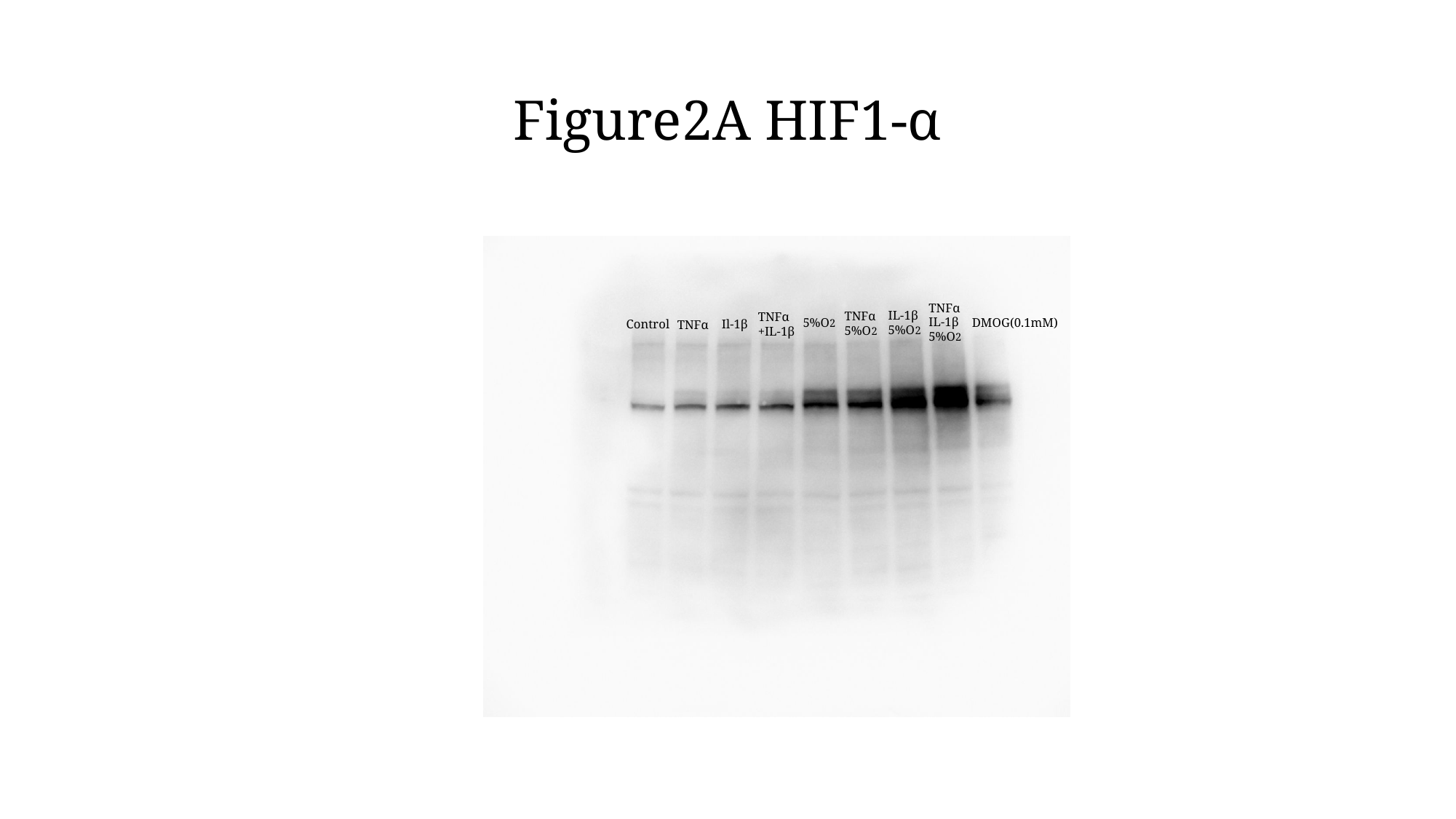

# Figure2A HIF1-α
TNFα
IL-1β
5%O2
IL-1β
5%O2
TNFα
5%O2
TNFα
+IL-1β
5%O2
DMOG(0.1mM)
Control
Il-1β
TNFα

## Slide 6
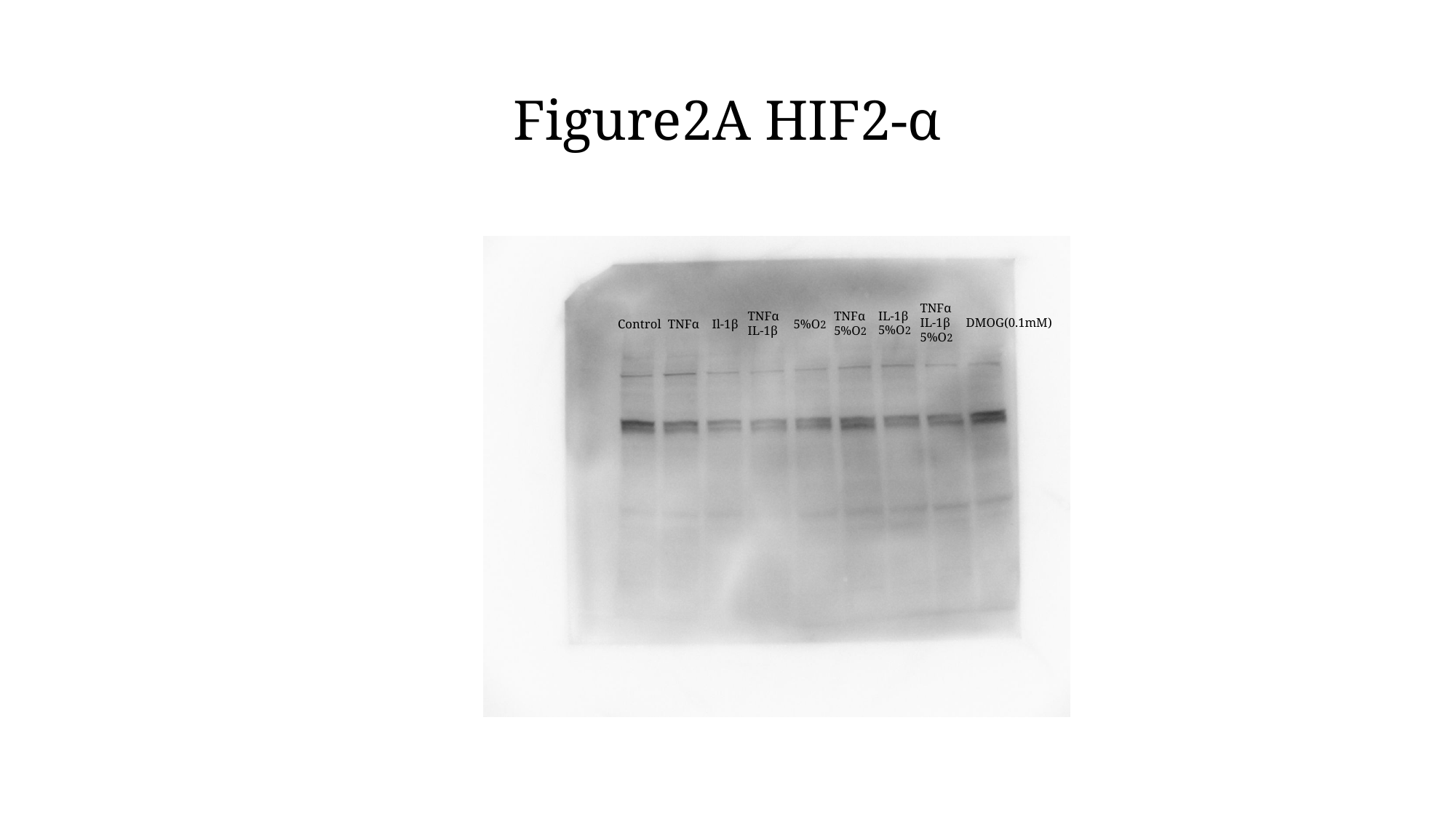

# Figure2A HIF2-α
TNFα
IL-1β
5%O2
IL-1β
5%O2
TNFα
IL-1β
TNFα
5%O2
DMOG(0.1mM)
Il-1β
5%O2
Control
TNFα

## Slide 7
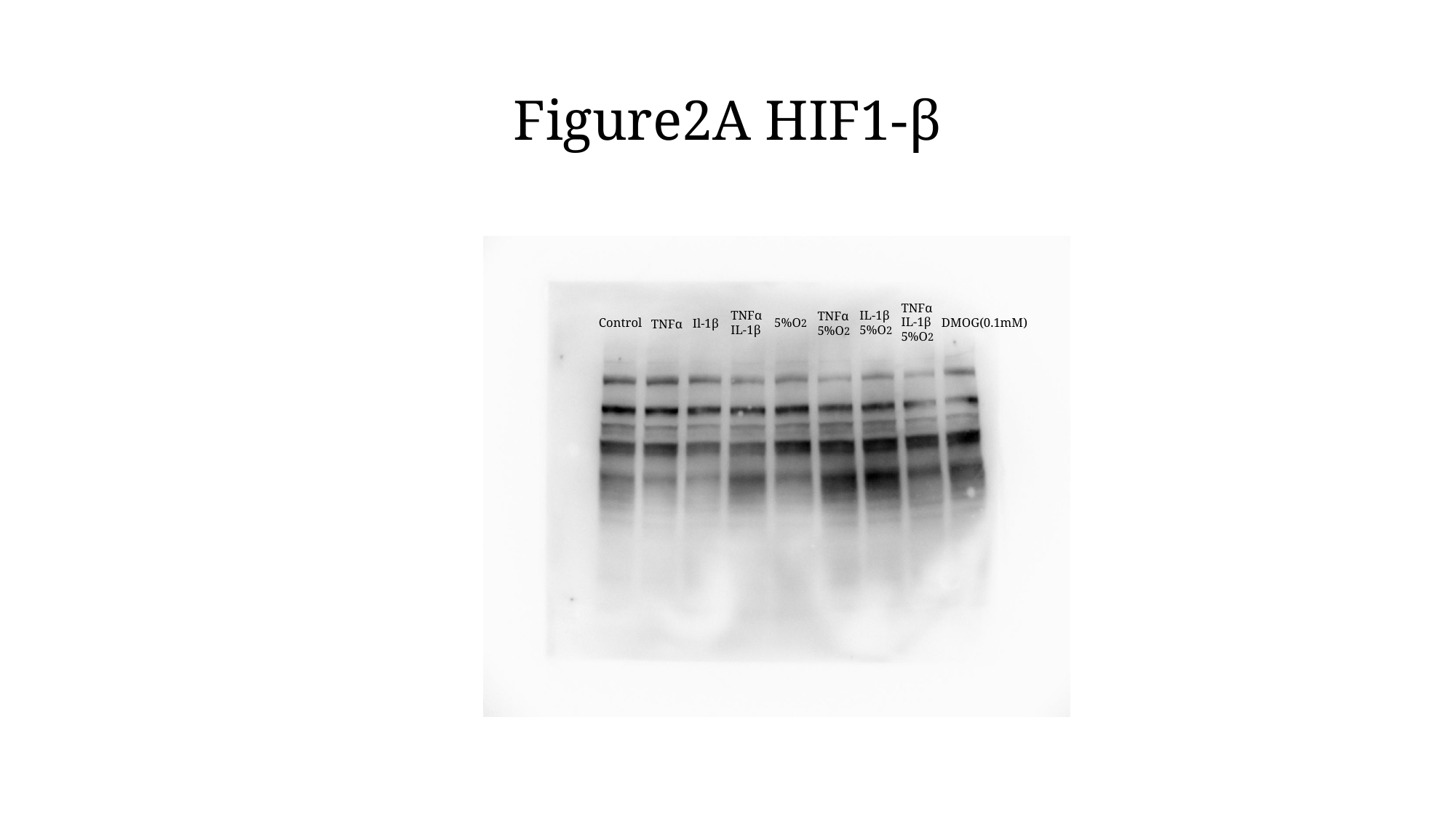

# Figure2A HIF1-β
TNFα
IL-1β
5%O2
TNFα
IL-1β
IL-1β
5%O2
TNFα
5%O2
Control
5%O2
DMOG(0.1mM)
Il-1β
TNFα

## Slide 8
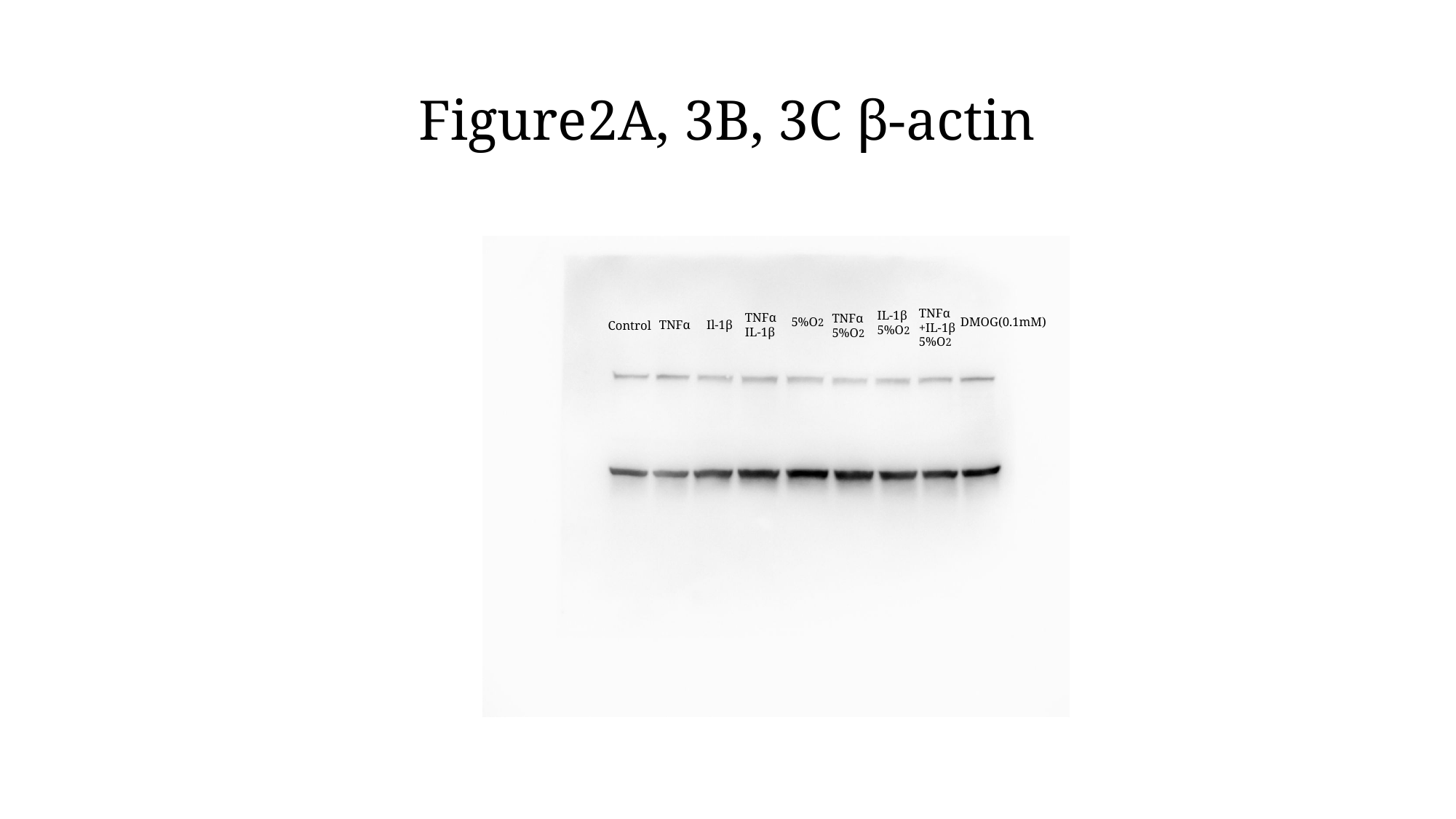

# Figure2A, 3B, 3C β-actin
TNFα
+IL-1β
5%O2
IL-1β
5%O2
TNFα
IL-1β
TNFα
5%O2
5%O2
DMOG(0.1mM)
TNFα
Il-1β
Control

## Slide 9
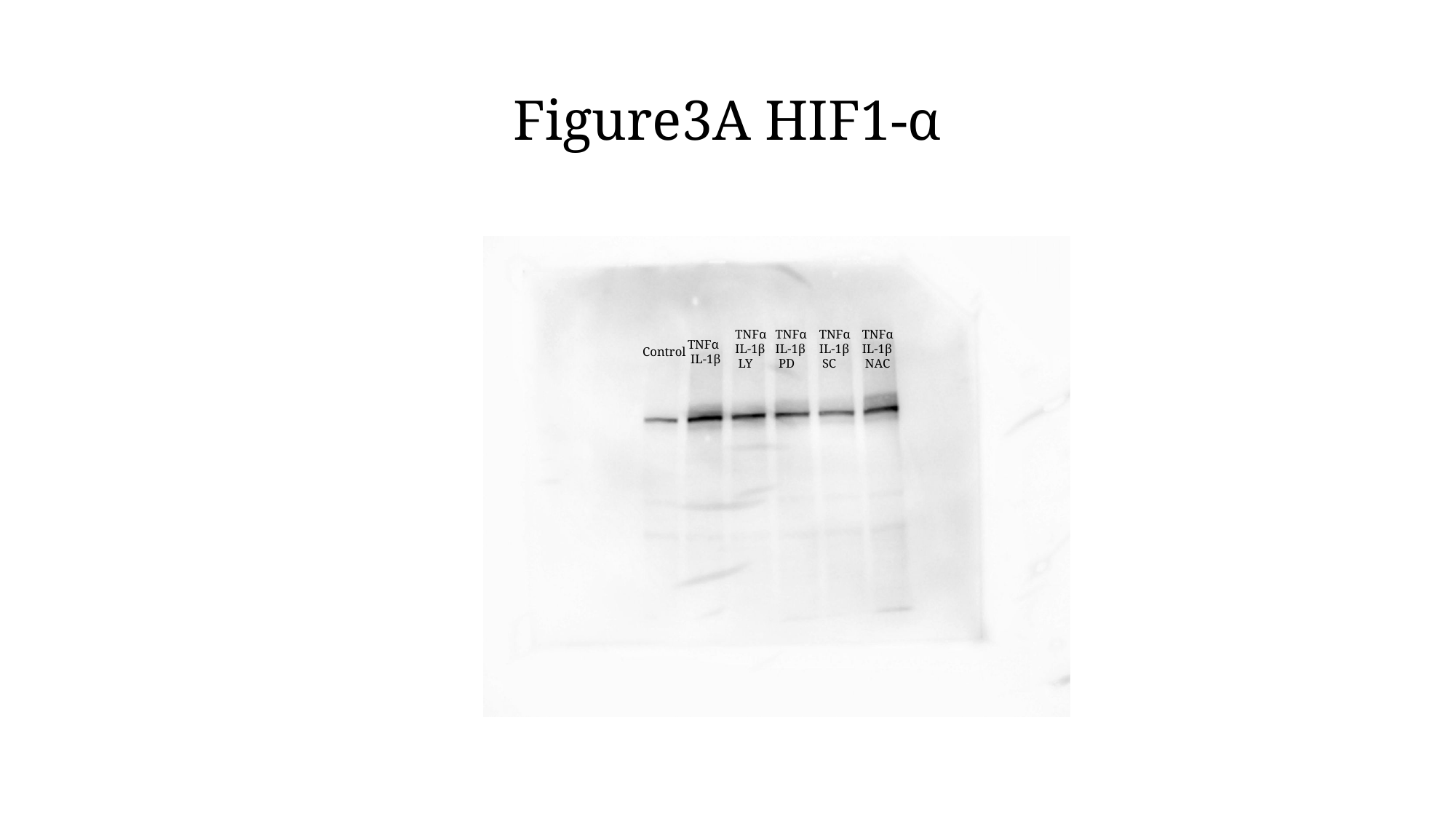

# Figure3A HIF1-α
TNFα
IL-1β
 LY
TNFα
IL-1β
 SC
TNFα
IL-1β
 NAC
TNFα
IL-1β
 PD
TNFα
 IL-1β
Control

## Slide 10
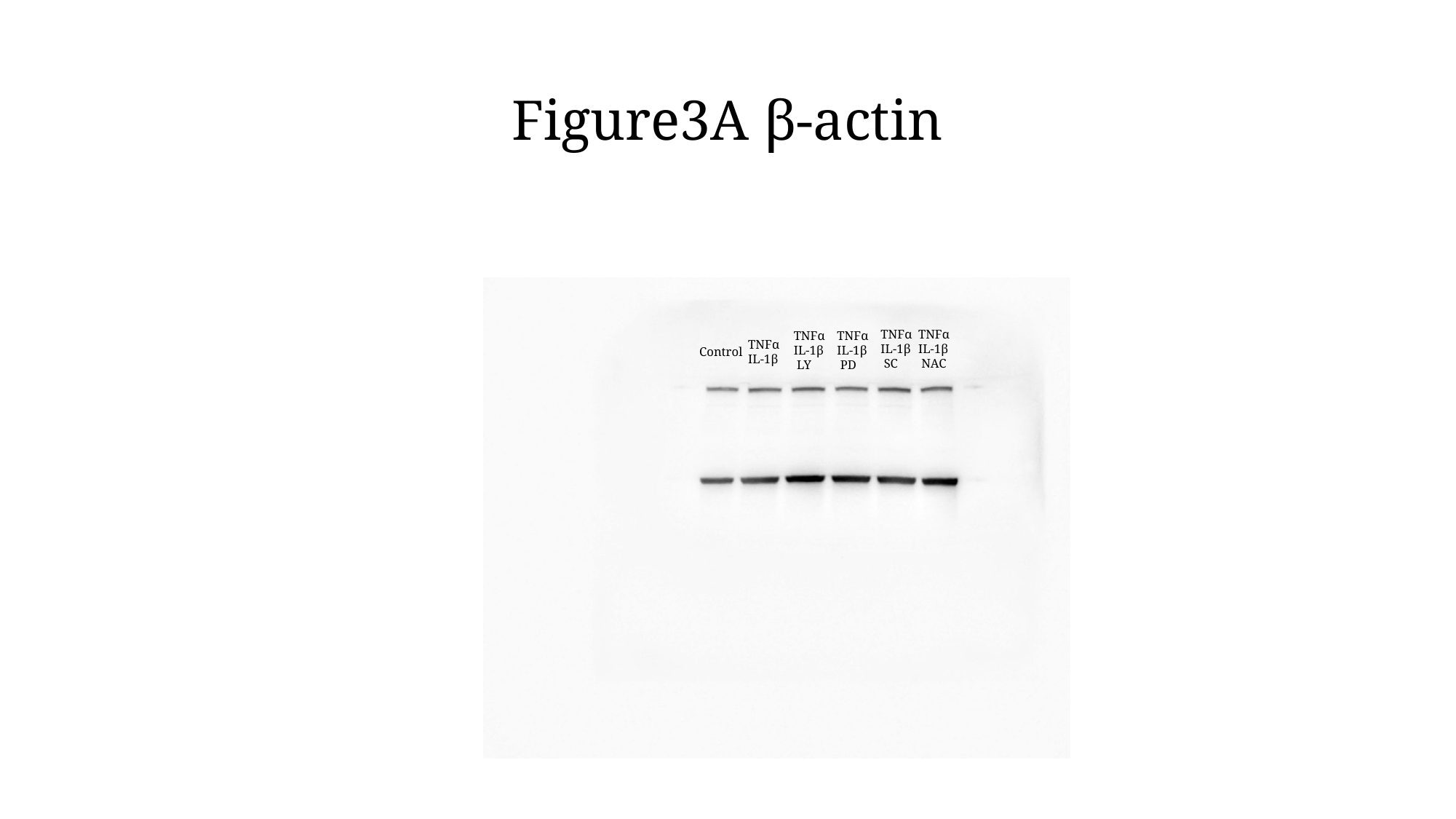

# Figure3A β-actin
TNFα
IL-1β
 SC
TNFα
IL-1β
 NAC
TNFα
IL-1β
 LY
TNFα
IL-1β
 PD
TNFα
IL-1β
Control

## Slide 11
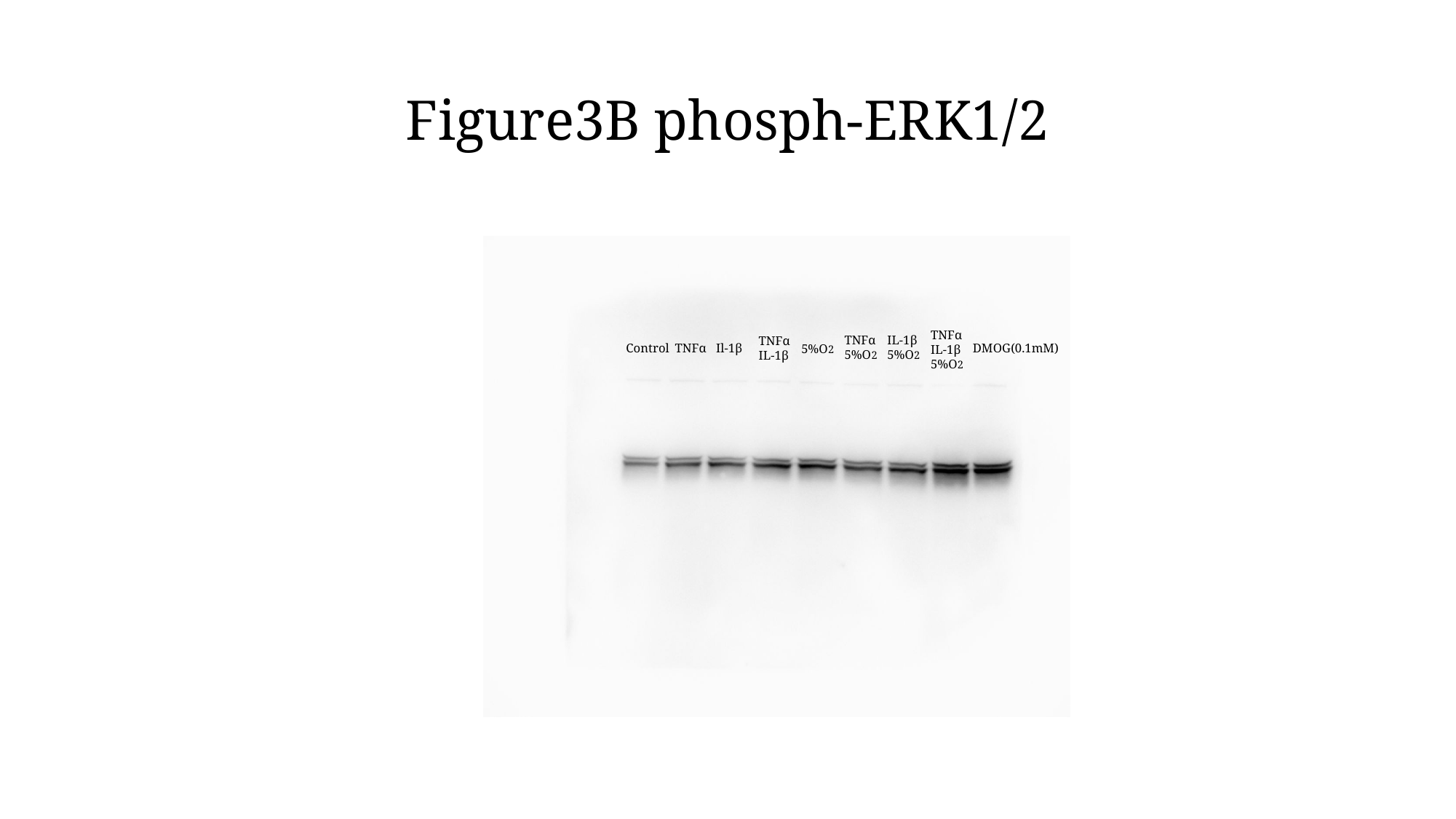

# Figure3B phosph-ERK1/2
TNFα
IL-1β
5%O2
TNFα
5%O2
IL-1β
5%O2
TNFα
IL-1β
Control
TNFα
Il-1β
DMOG(0.1mM)
5%O2

## Slide 12
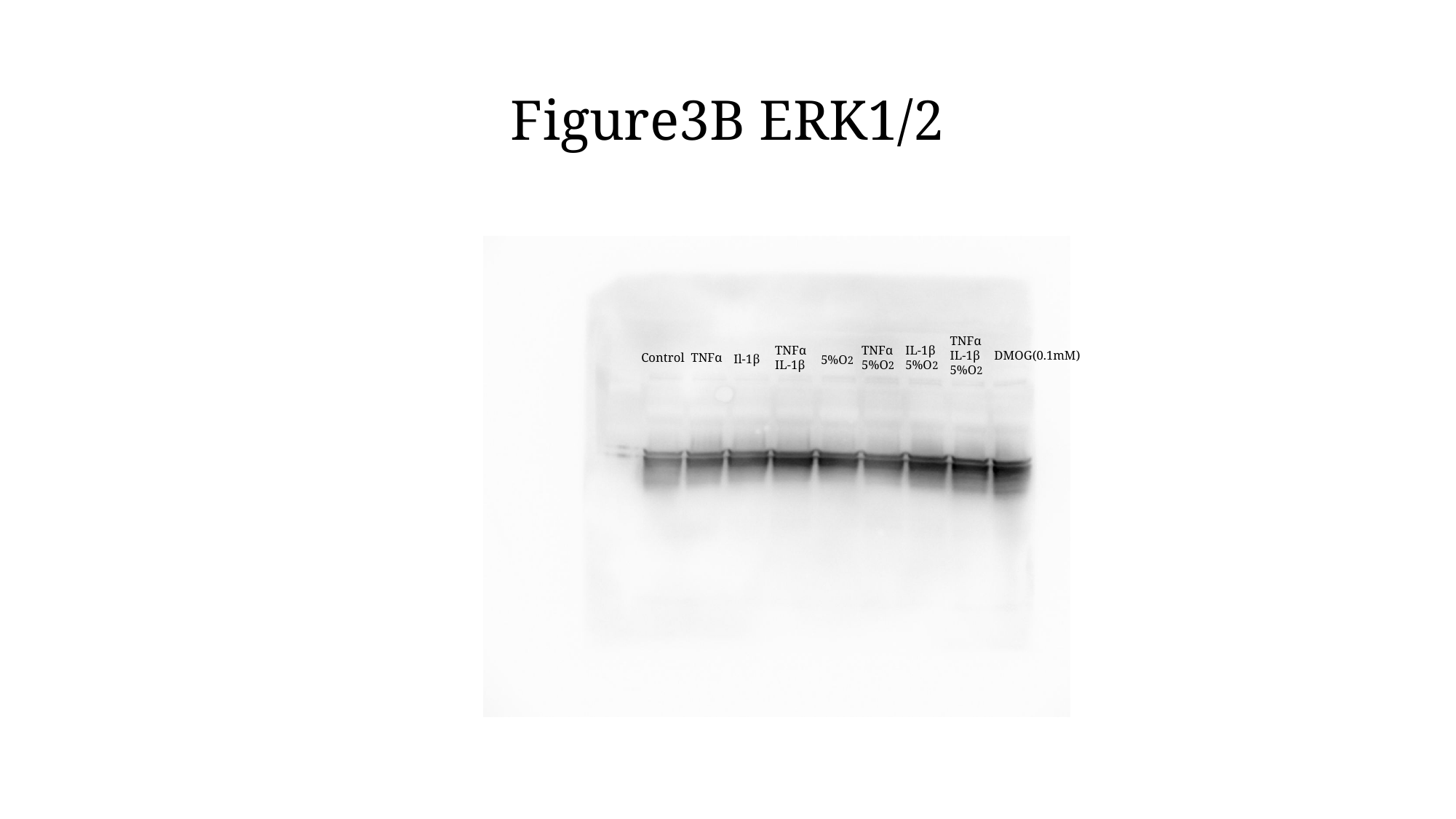

# Figure3B ERK1/2
TNFα
IL-1β
5%O2
TNFα
IL-1β
TNFα
5%O2
IL-1β
5%O2
DMOG(0.1mM)
Control
TNFα
Il-1β
5%O2

## Slide 13
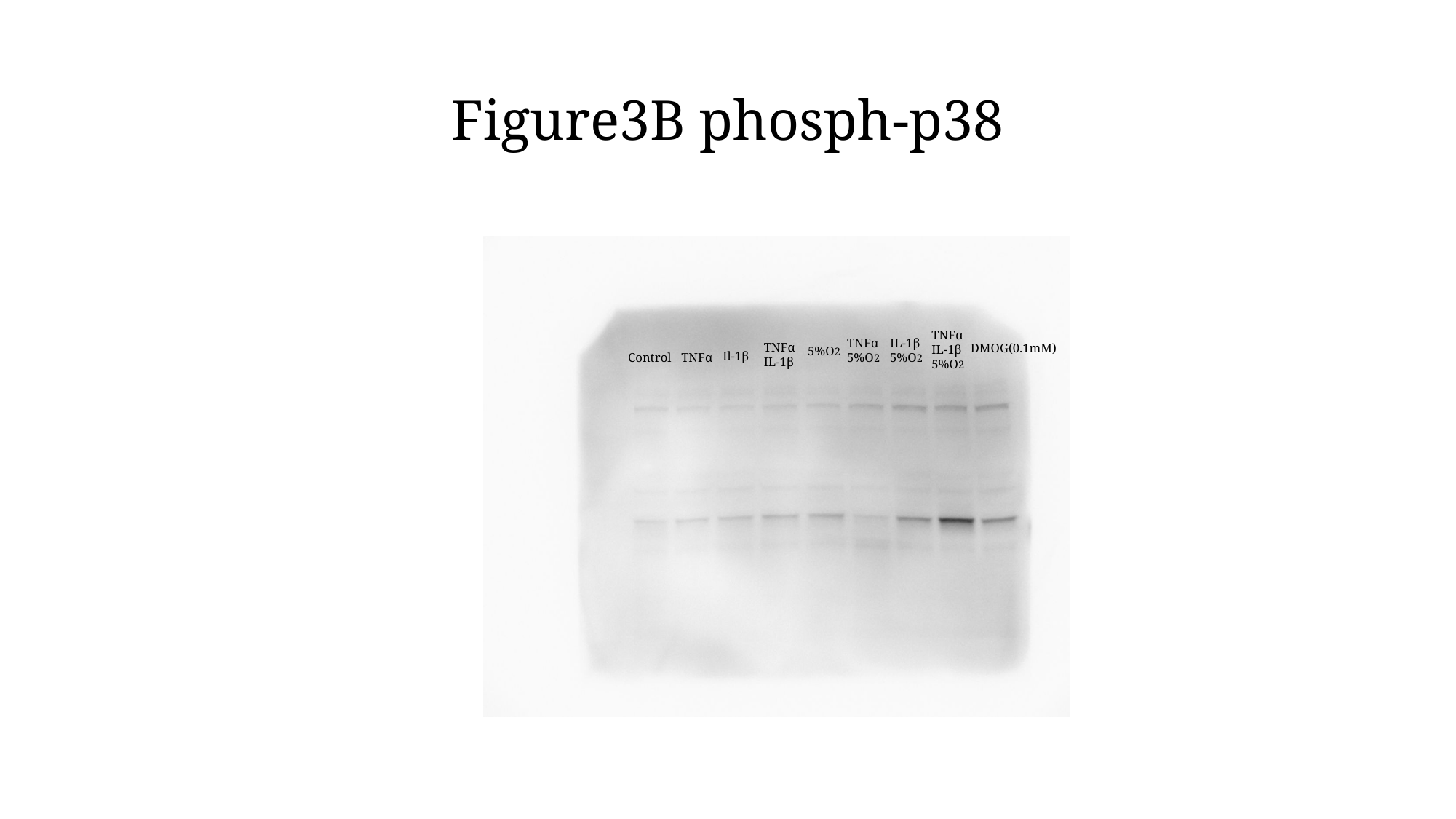

# Figure3B phosph-p38
TNFα
IL-1β
5%O2
IL-1β
5%O2
TNFα
5%O2
TNFα
IL-1β
DMOG(0.1mM)
5%O2
Il-1β
Control
TNFα

## Slide 14
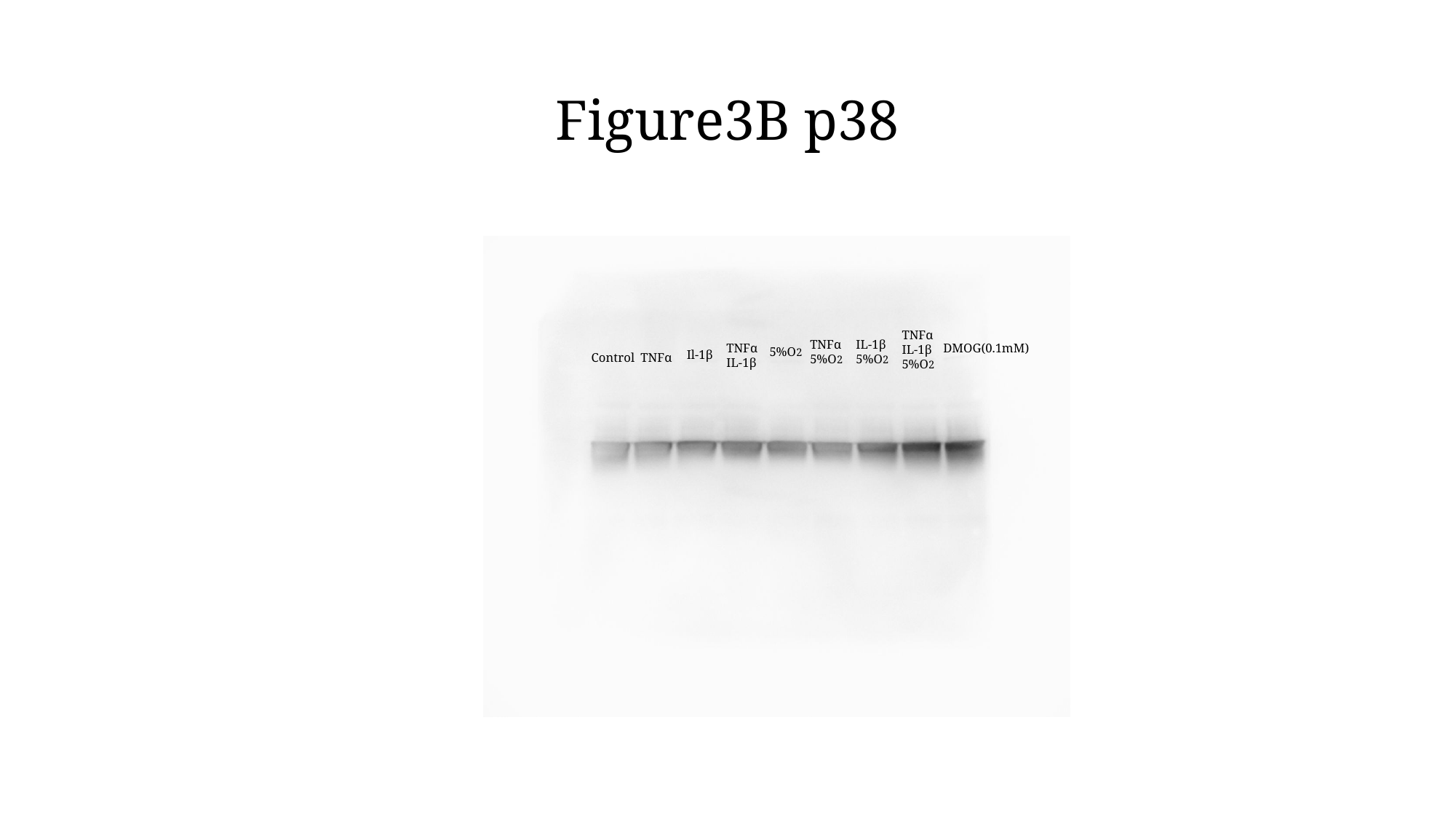

# Figure3B p38
TNFα
IL-1β
5%O2
TNFα
5%O2
IL-1β
5%O2
TNFα
IL-1β
DMOG(0.1mM)
5%O2
Il-1β
Control
TNFα

## Slide 15
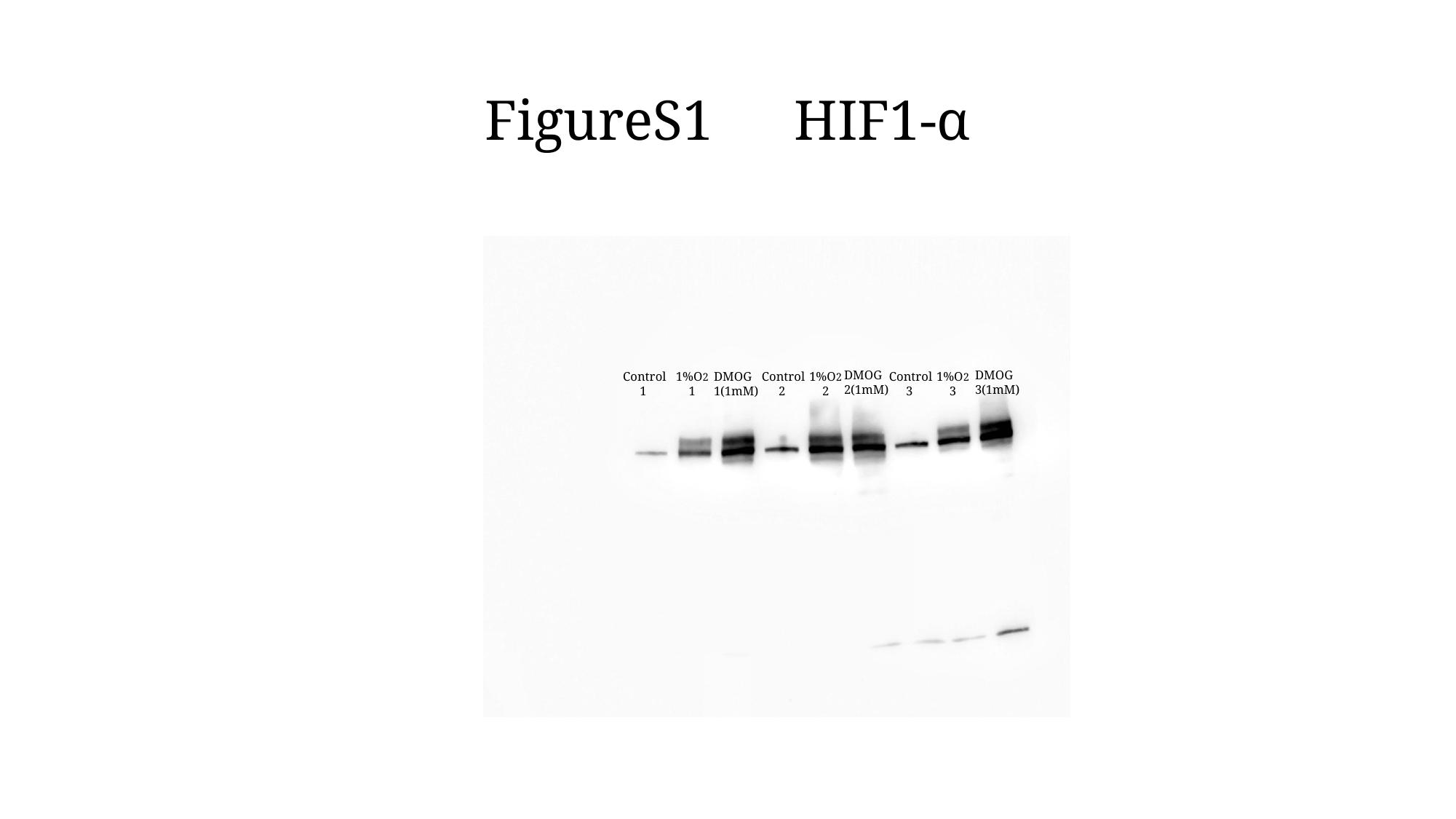

# FigureS1　HIF1-α
DMOG
2(1mM)
DMOG
3(1mM)
Control
1
1%O2
1
DMOG
1(1mM)
Control
2
Control
3
1%O2
3
1%O2
2

## Slide 16
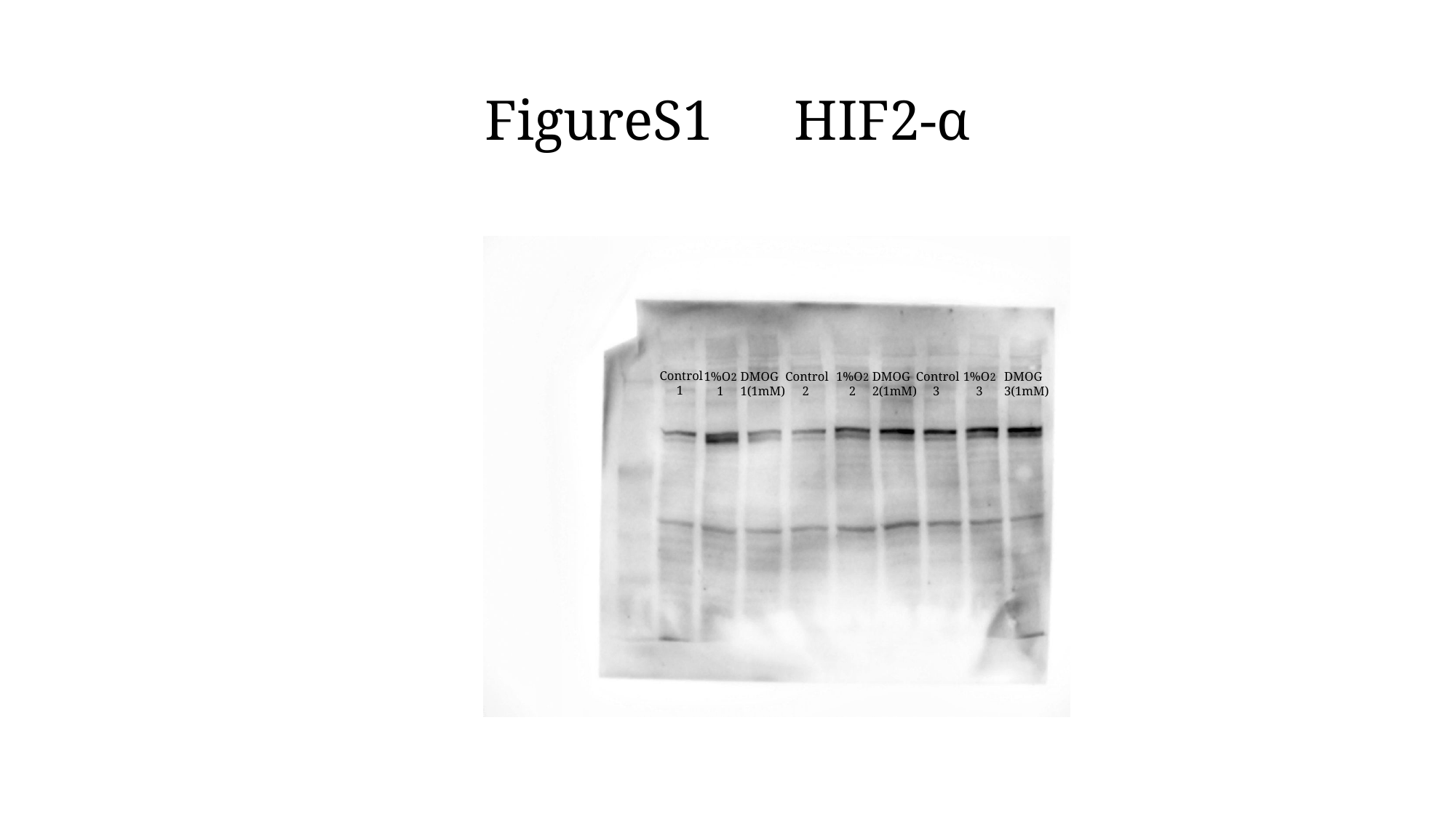

# FigureS1　HIF2-α
Control
1
Control
2
1%O2
2
DMOG
2(1mM)
Control
3
1%O2
3
DMOG
3(1mM)
1%O2
1
DMOG
1(1mM)

## Slide 17
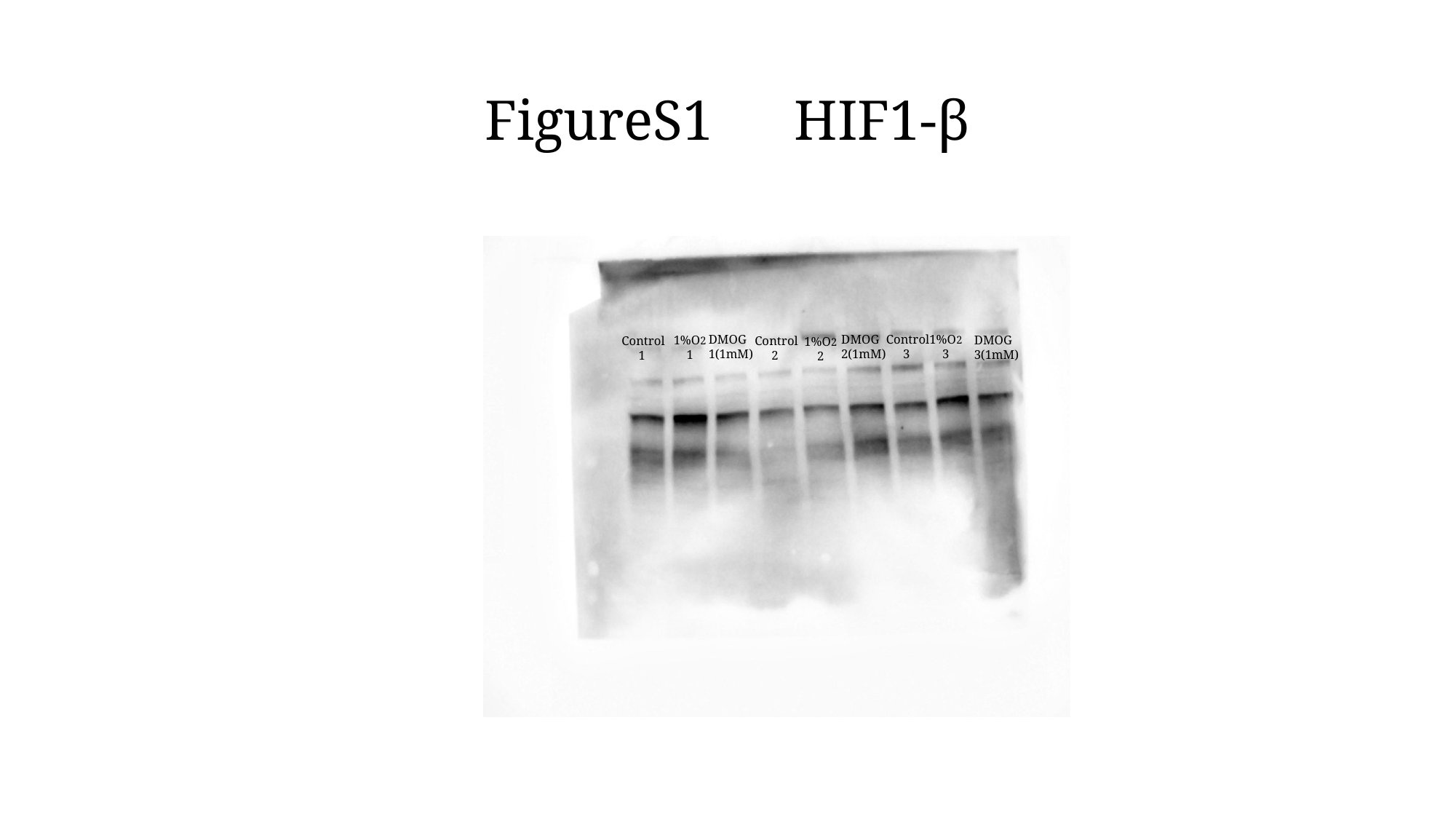

# FigureS1　HIF1-β
DMOG
1(1mM)
DMOG
2(1mM)
Control
3
1%O2
3
DMOG
3(1mM)
1%O2
1
Control
1
Control
2
1%O2
2

## Slide 18
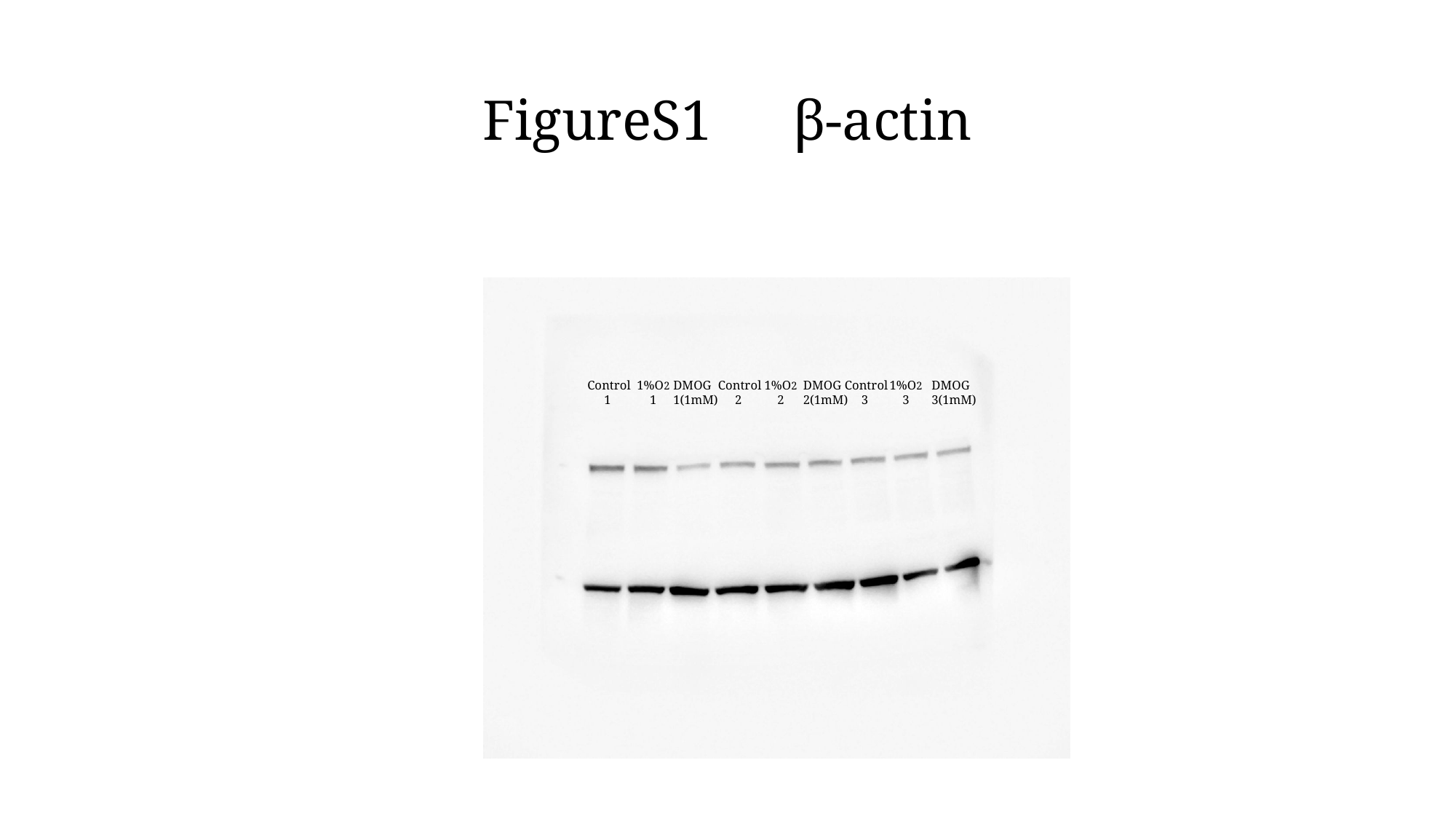

# FigureS1　β-actin
Control
1
DMOG
1(1mM)
Control
2
DMOG
2(1mM)
Control
3
1%O2
3
DMOG
3(1mM)
1%O2
1
1%O2
2
