# Supplementary material for: Inflammatory Cytokine-Induced HIF-1 Activation Promotes Epithelial–Mesenchymal Transition in Endometrial Epithelial Cells
Source: Biomedicines. 2023 Jan 14;11(1):210. doi: 10.3390/biomedicines11010210 (PMC9855875; doi:10.3390/biomedicines11010210)
Supplement: Supplementary file 1 [file biomedicines-11-00210-s001.zip › supplementary_captions_2.pdf]

Table S1. The expression matrix, calculated as scaled TPM and loaded at gene level by tximport. e_ctl: control, e_cyt: treated by cytokines (500 ng/mL TNFα and 100 ng/mL IL-1β) under 20% O_2 conditions for 8h, e_hypo: exposed to 1% O_2 for 8h.

Table S2. Gene set enrichment analyses for gene lists analyzed by RNA-Seq were performed using Metascape database between cytokines (500 ng/mL TNFα and 100 ng/mL IL-1β)-treatment between control groups.

Table S3. Gene set enrichment analyses for gene lists analyzed by RNA-Seq were performed using Metascape database between 1% O_2 conditions between control groups.

Figure S1. Under hypoxic situations, pro-inflammatory substances cause the buildup of HIF-1 protein in endometrial epithelial cells. Immortalized endometrial epithelial cells EM-E6/E7/TERT were incubated with culture media (-) or DMOG (1 mmol/L) under 20% or 1% O2 conditions for 6 h before protein were extracted. (A) Illustrations of immunoblots. Immunoblotting was done on whole-cell lysates for HIF-1α, HIF-2α, HIF-1β, and β-actin. There were at least three such experiments carried out. (B) Densitometric analysis results that have been β-actin normalized.

Figure S2. Principal component analysis (PCA) for the RNA-Seq results were performed using iDEP 0.93.

Figure S3. Results of gene set enrichment analyses with the gene sets whose expression was upregulated by pro-inflammatory cytokine treatment (500 ng/mL TNFα and 100 ng/mL IL-1β).

Figure S4. Results of gene set enrichment analyses with the gene sets whose expression was upregulated by exposure to 1% O_2.

Figure S5. Results of gene set enrichment analyses with the gene sets whose expression was commonly upregulated by pro-inflammatory cytokine treatment (500 ng/mL TNFα and 100 ng/mL IL-1β) and exposure to 1% O_2.
