# Supplementary figures and images for: Inflammatory Cytokine-Induced HIF-1 Activation Promotes Epithelial–Mesenchymal Transition in Endometrial Epithelial Cells
Source: Biomedicines. 2023 Jan 14;11(1):210. doi: 10.3390/biomedicines11010210 (PMC9855875; doi:10.3390/biomedicines11010210)

**A**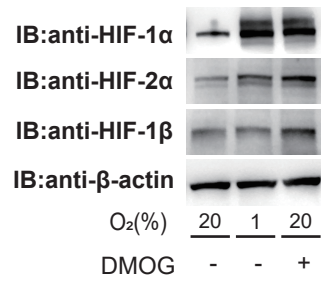**B**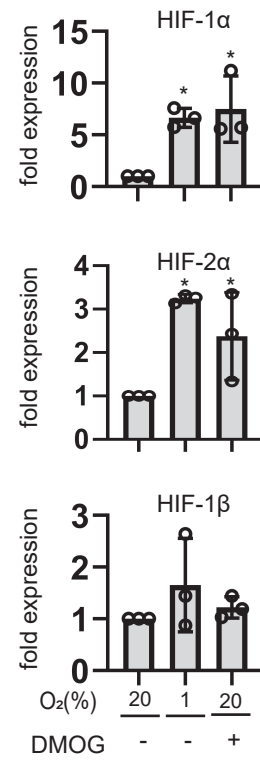

Supplement: Supplementary file 1 [file biomedicines-11-00210-s001.zip › figure_S1.pdf]

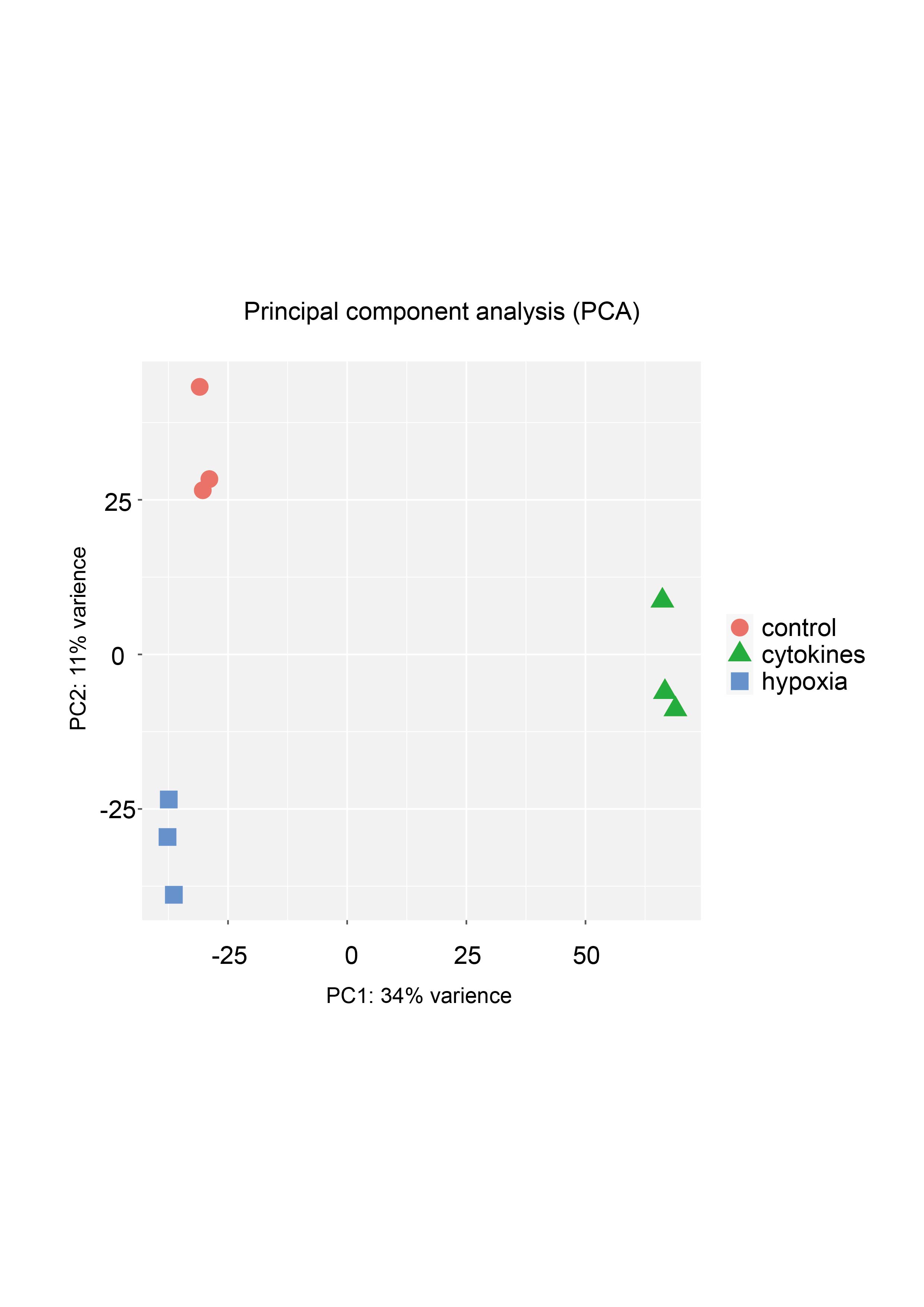

Supplement: Supplementary file 1 [file biomedicines-11-00210-s001.zip › figure_S2.jpg]

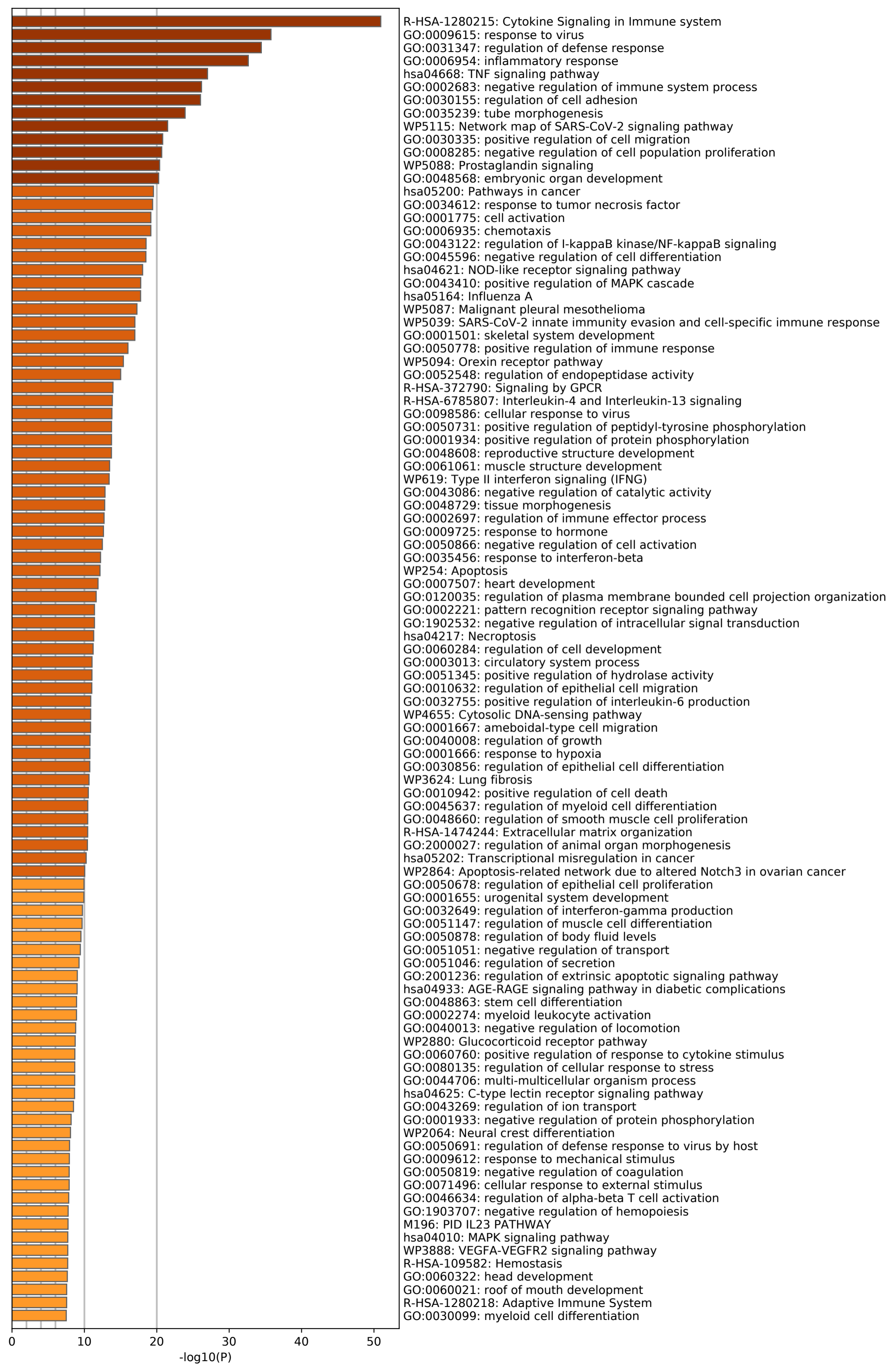

Supplement: Supplementary file 1 [file biomedicines-11-00210-s001.zip › figure_S3.pdf]

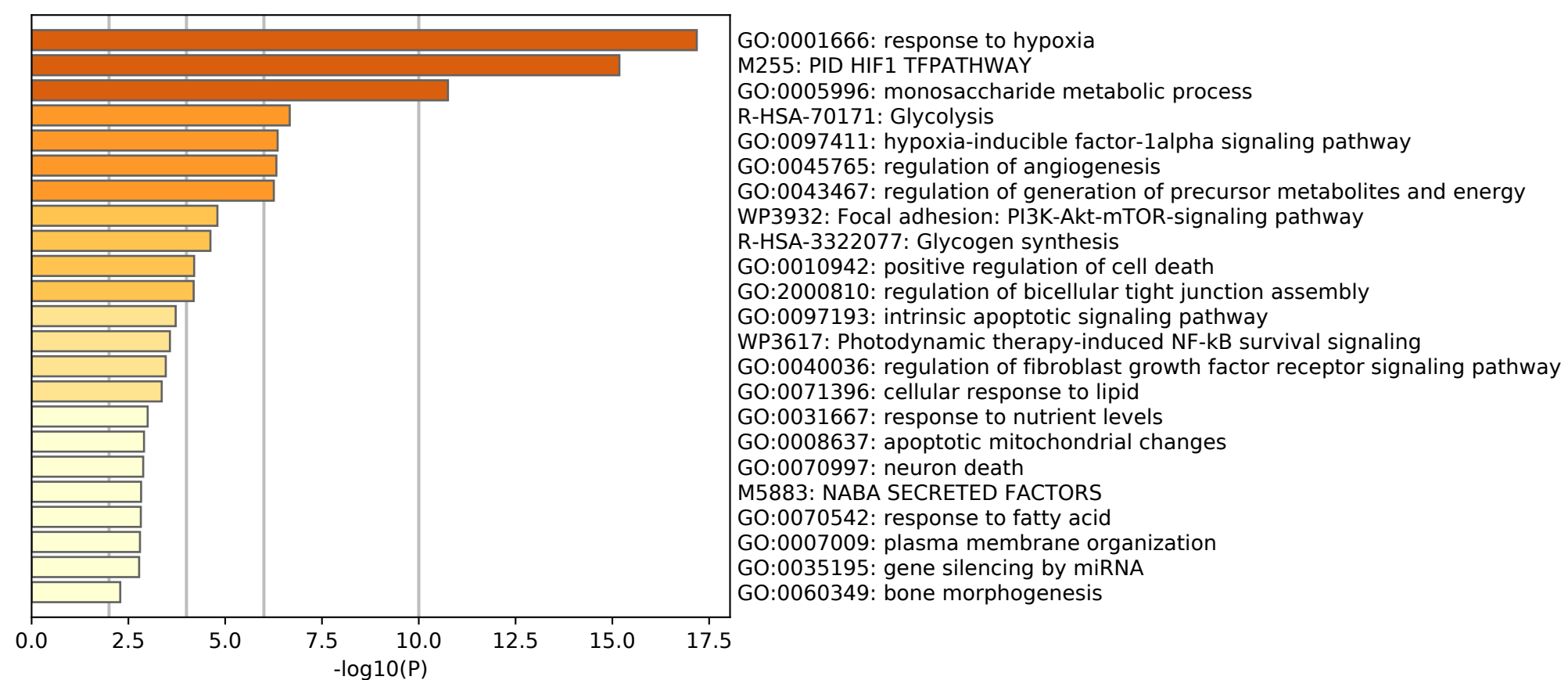

Supplement: Supplementary file 1 [file biomedicines-11-00210-s001.zip › figure_S4.pdf]

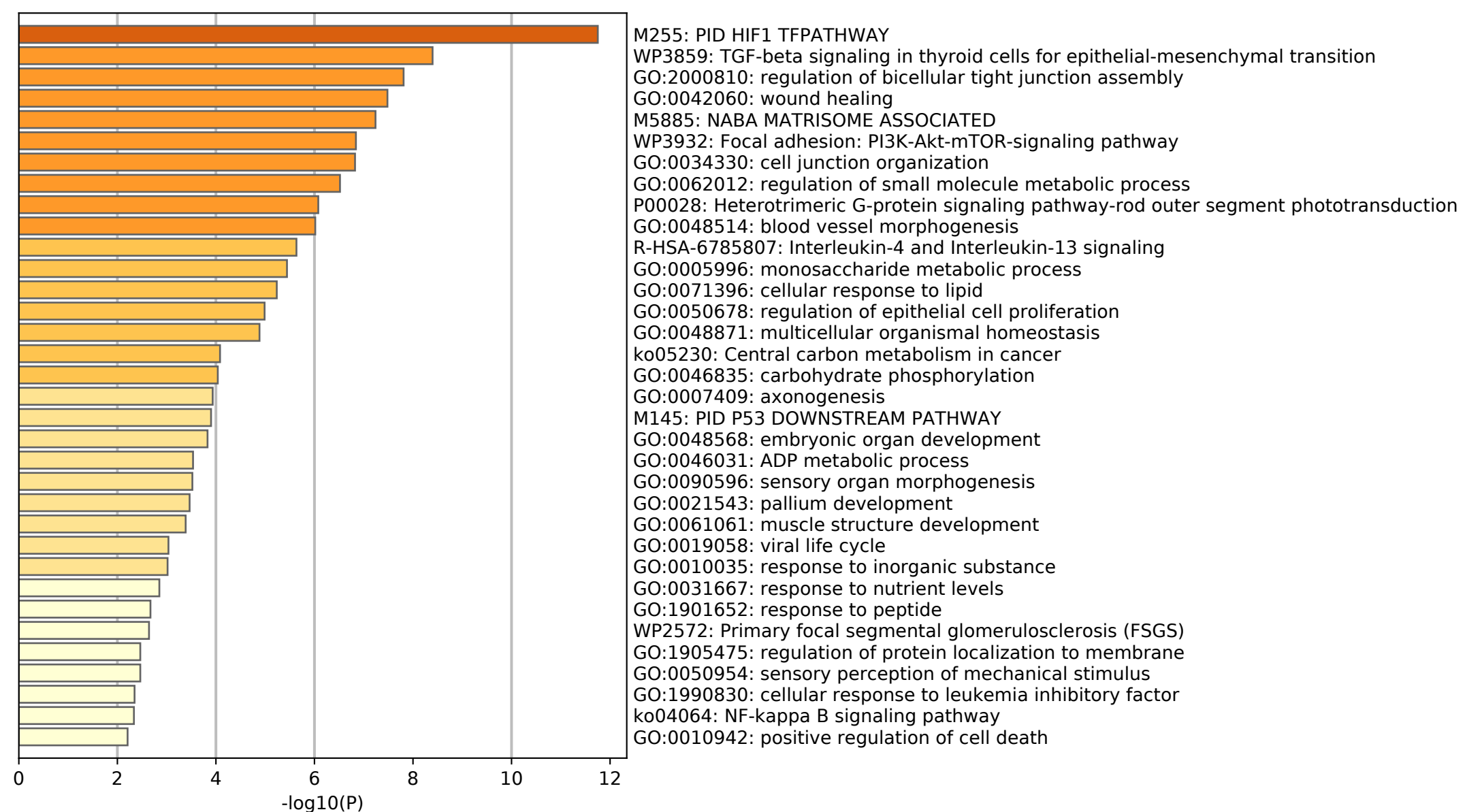

Supplement: Supplementary file 1 [file biomedicines-11-00210-s001.zip › figure_S5.pdf]
